# Elevated expression of the rhythm gene *NFIL3* promotes the progression of TNBC by activating NF-κB signaling through suppression of *NFKBIA* transcription

**DOI:** 10.1186/s13046-022-02260-1

**Published:** 2022-02-18

**Authors:** Weiwei Yang, Jing Li, Minghui Zhang, Haichuan Yu, Yuan Zhuang, Lingyu Zhao, Lili Ren, Jinan Gong, Hongjie Bi, Lixuan Zeng, Yang Xue, Jinjin Yang, Yan Zhao, Shuoshuo Wang, Shuangshu Gao, Zitong Fu, Dongze Li, Jinxing Zhang, Tianzhen Wang, Ming Shan, Bo Tang, Xiaobo Li

**Affiliations:** 1 Department of Pathology, Harbin Medical University, Harbin, China; 2 Electronic Microscope Center of Harbin Medical University, Harbin, China; 3 Department of Oncology, Chifeng City Hospital, Chifeng, China; 4 School of Medical Laboratory, Xinxiang Medical University, Xinxiang, China; 5 Clinicopathological Diagnosis Center, Qiqihar Medical University, Qiqihar, China; 6 Department of Breast Surgery, the Affiliated Tumor Hospital of Harbin Medical University, Harbin, China; 7 Department of Hepatobiliary Surgery, the First Affiliated Hospital of Guangxi Medical University, Nanning, China

**Keywords:** Rhythm genes, NFIL3, Triple-negative breast cancer, Tumor associated inflammation

## Abstract

**Background:**

Epidemiological studies have confirmed that abnormal circadian rhythms are associated with tumorigenesis in breast cancer. However, few studies have investigated the pathological roles of rhythm genes in breast cancer progression. In this study, we aimed to evaluate the aberrant expression of 32 rhythm genes in breast cancer and detect the pathological roles and molecular mechanisms of the altered rhythm gene in regulating the progression of triple negative breast cancer (TNBC).

**Methods:**

The aberrant expression of rhythm genes in breast cancer was screened by searching the GEPIA database and validated by using qRT-PCR and immunohistochemistry staining. Bioinformatics analysis combined with luciferase reporter experiment and chromatinimmunopercitation (ChIP) were used to investigate the molecular mechanism about aberrant expression of identified rhythm gene in breast cancer. The pathological roles of identified rhythm gene in TNBC progression was evaluated by colony formation assay, wound healing experiment, transwell assay, subcutaneous tumor formation and the mouse tail vein injection model through gain-of-function and loss-of-function strategies respectively. mRNA array, bioinformatics analysis, luciferase reporter experiment, ChIP and immunoflurescence assay were employed to investigate the key molecules and signaling pathways by which the identified rhythm gene regulating TNBC progression.

**Results:**

We identified that nuclear factor interleukin 3 regulated (NFIL3) expression is significantly altered in TNBC compared with both normal breast tissues and other subtypes of breast cancer. We found that NFIL3 inhibits its own transcription, and thus, downregulated NFIL3 mRNA indicates high expression of NFIL3 protein in breast cancer. We demonstrated that NFIL3 promotes the proliferation and metastasis of TNBC cells in vitro and in vivo, and higher expression of NFIL3 is associated with poor prognosis of patients with TNBC. We further demonstrated that NFIL3 enhances the activity of NF-κB signaling. Mechanistically, we revealed that NFIL3 directly suppresses the transcription of NFKBIA, which blocks the activation of NF-κB and inhibits the progression of TNBC cells in vitro and in vivo. Moreover, we showed that enhancing NF-κB activity by repressing NFKBIA largely mimics the oncogenic effect of NFIL3 in TNBC, and anti-inflammatory strategies targeting NF-κB activity block the oncogenic roles of NFIL3 in TNBC.

**Conclusion:**

NFIL3 promotes the progression of TNBC by suppressing *NFKBIA* transcription and then enhancing NF-κB signaling-mediated cancer-associated inflammation. This study may provide a new target for TNBC prevention and therapy.

**Graphical Abstract:**

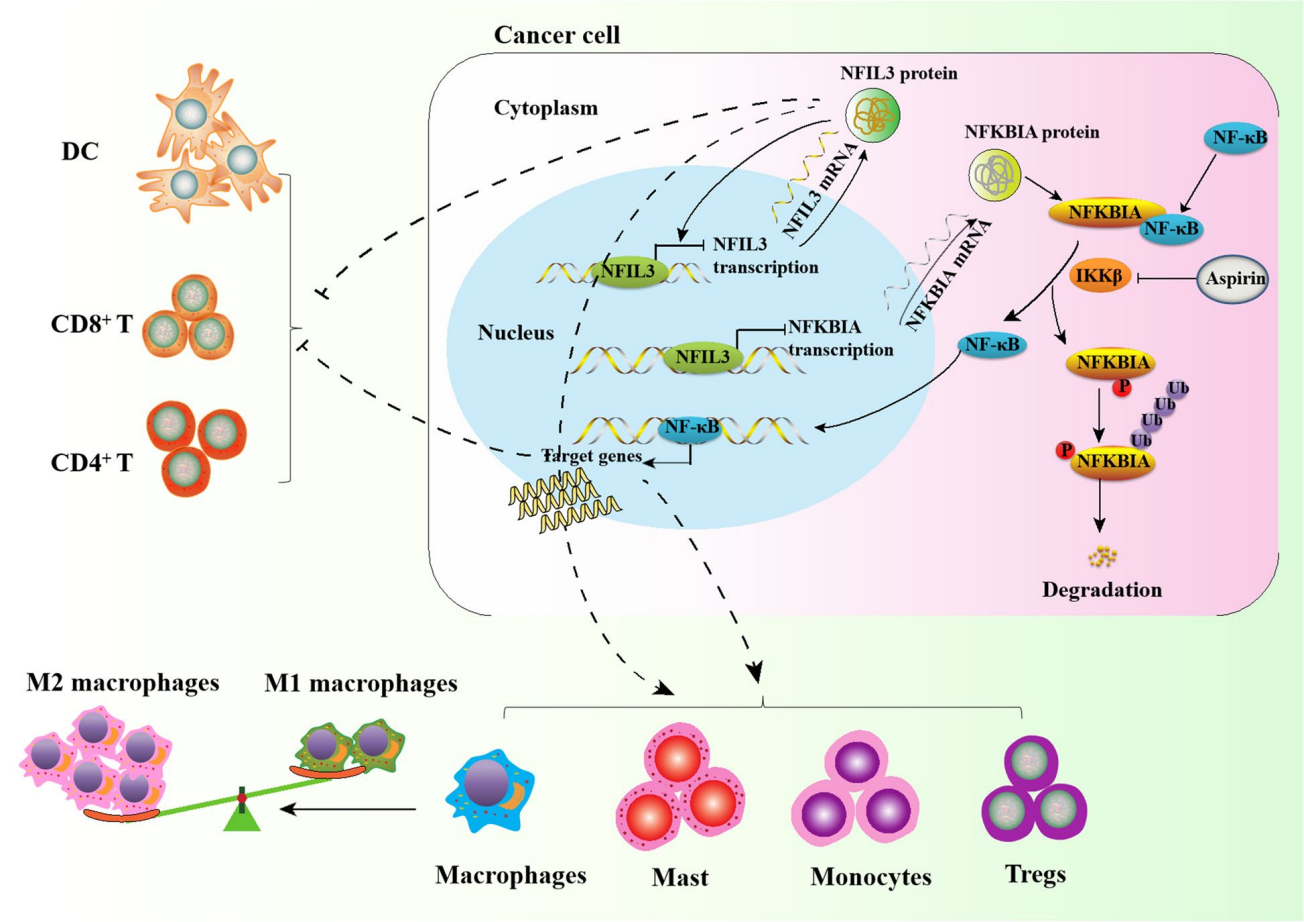

**Supplementary Information:**

The online version contains supplementary material available at 10.1186/s13046-022-02260-1.

## Background

Circadian rhythm genes not only regulate the circadian rhythm [1] but also regulate physiological activities and behavioral patterns in humans, such as sleep patterns, hormone release, feeding behaviors, body temperature and blood pressure [2]. Recent studies have shown that abnormal circadian rhythms are associated with numerous diseases, including cancer [3].

Breast cancer is one of the most common cancers in females and the second leading cause of cancer-related deaths among women [4]. Although overall survival is significantly improved in early-stage breast cancers because of the innovation of endocrine therapy and targeted therapy, some patients with triple-negative breast cancer (TNBC) still have a poor prognosis due to a lack of clear molecular mechanisms and effective therapeutic targets.

Recent evidence has shown that alterations in circadian rhythms or rhythm genes are associated with susceptibility to breast cancer. Epidemiological studies have indicated that abnormal circadian rhythms enhance the risk of breast cancer [5]. Additionally, polymorphisms of rhythm genes, including BMAL1, CRY2, PER1, PER2 and PER3, are associated with susceptibility to breast cancer [6]. Moreover, the abnormal expression of rhythm genes was also associated with the occurrence and progression of breast cancer [7]. For example, downregulation of the circadian gene PER2 increases Cyclin D and Cyclin E levels and consequently accelerates breast cancer growth [8]. The expression levels of rhythm genes, such as CLOCK, PER1, PER2, PER3, CRY2, RORC and TIMELESS, are associated with the metastasis-free survival of breast cancer patients [9].

Although several rhythm genes are reportedly associated with the progression and prognosis of breast cancer, the functions and mechanisms of most rhythm genes in breast cancer are undetermined. We previously identified 32 rhythm genes and analyzed their expression alterations across cancers and revealed five rhythm genes, including nuclear factor interleukin 3 regulated (NFIL3), that were abnormally expressed in most of the detected cancer types [10]. In this study, we evaluated the aberrant expression of 32 rhythm genes in breast cancer and identified that the expression of NFIL3 was not only altered in breast cancer tissues compared with normal breast tissues but also significantly different between TNBC and other subtypes of breast cancer. We also examined the roles of NFIL3 in regulating the proliferation and metastasis of TNBC and explored the potential mechanisms by which NFIL3 regulates the progression of TNBC. This study may not only elaborate the molecular mechanisms by which NFIL3 regulates cancer progression but also provide a new target for TNBC therapy.

## Material and methods

### Cell culture and reagents

Two triple-negative breast cancer cell lines, Hs578T and BT549, human colorectal adenocarcinoma cell line HCT15, normal breast epithelial cells MCF10A, human lung cancer cells A549, ovarian cancer cells SKOV3, human cervical cancer cells HELA, human pancreatic cancer cells PANC-1 were all purchased from Cell Lines Service (Procell Co., Ltd., Wuhan, China) and authenticated by STR (short tandem repeats). Human normal colonic epithelial cells NCM460 and breast cancer cells HCC70 were purchased from Shanghai Binsui Biotechnology Co., Ltd. Human laryngeal carcinoma cells TU212 and human oral cancer cells TU138 were purchased from Shanghai Xinyu Biotechnology Co., Ltd. 293 T cells used for lentiviral vector packaging were purchased from the American Type Culture Collection (ATCC). These cells were cultured in DMEM (Gibco, NY, USA) supplemented with 10% fetal bovine serum (Gibco), 100 IU/mL penicillin and 100 μg/mL streptomycin at 37 °C in a humidified incubator containing 5% CO_2_. Before an experimental run, Mycoplasma testing was performed (MycoAlert™, Lonza).

Aspirin was purchased from Sigma (Sigma–Aldrich, St. Louis, MO, USA) and dissolved in ethanol. The specific NF-κB inhibitor Bay11–7082 was purchased from Calbiochem (Calbiochem, San Diego, CA, USA) and dissolved in dimethylsulfoxide (DMSO, Solarbio Life Sciences, Beijing, China) as the storage solution. Recombinant murine TNF-α was purchased from PeproTech (Rocky Hill, NJ, USA) and dissolved in 0.1% BSA as the storage solution (100 μg/mL). Cycloheximide was purchased from MCE (MedChemexpress, Shanghai, China).

### Patients and specimens

A total of 120 paraffin-embedded breast cancer tissue samples were collected from the Department of Pathology at Chifeng City Hospital, 20 noncancerous breast tissue samples, 20 lung squamous cell carcinoma tissues, 20 lung adenocarcinoma tissues, 20 cervical cancer tissues, 20 ovarian cancer tissues and 20 corresponding normal tissues were collected after surgical removal from the Department of Pathology at Harbin Medical University Cancer Hospital. Additionally, the paraffin-embedded TNBC tissues from 60 patients with more than 5 years of postoperative follow-up records were also collected from the Department of Pathology at Harbin Medical University Cancer Hospital. Written informed consent was obtained from each patient, and the study was approved by the institution’s ethics committee.

### Bioinformatics analysis

The GEPIA database was used to analyze gene expression based on data from the TCGA and GTEx databases (http://gepia.cancer-pku.cn/index.html) [11]. The expression of NFIL3 and other rhythm genes in pancancer or breast cancer and normal samples was obtained using GEPIA. In addition, the relationship between NFIL3 and NFKBIA was also analyzed by GEPIA. Cistrome Data Browser (http://cistrome.org/db/#/) was used to screen the DNA binding sites of NFIL3 in its target genes. The correlation between the infiltration of 22 inflammatory cells and NFIL3 mRNA levels or NFKBIA mRNA levels were calculated by the Pearson correlation coefficient as previously described through analyzing the mRNA expression data of breast cancer tissues from the TCGA [12].

### Construction of plasmids and lentiviral vector packaging

The putative NFIL3-binding sites in the promoter of the NFIL3 or NFKBIA gene were amplified by PCR and then inserted into the PGL3-basic luciferase vector to generate luciferase reporter constructs. The sequences of the primers are shown in Supplementary Table 1.

Full-length NFIL3 and NFKBIA cDNA was synthesized and inserted into the pLVX plasmid to overexpress these genes. shRNA was used to knock down the expression of endogenous NFIL3 and NFKBIA, and the DNA fragments encoding the shRNA were synthesized and cloned into the pLKO.1 vector [13]. The sequence of the synthesized DNA fragment is shown in Supplementary Table 1. The lentiviral vectors were prepared and packaged as previously described [14]. Briefly, 293 T cells were grown to 80% confluence, and then the constructed plasmids were cotransfected with packaging plasmids pMD2.G and pSPAX2, respectively. After 24 h, the supernatants were collected and used to infect TNBC cells as previously described [15].

### Total RNA extraction and quantitative real-time PCR

Total RNA from cells was extracted using TRIzol reagent (Invitrogen, Carlsbad, CA, USA). Total RNA from paraffin tissue sections was extracted using an FFPE RNA kit (Omega Bio-Tek, Guangzhou, China) according to the manufacturer’s instructions. Total RNA was converted to cDNA by reverse transcription using the TransScript One-Step gDNA Removal and cDNA Synthesis SuperMix Kit (TransGen Biotech, Beijing, China). qRT–PCR was performed using the TransScript Tip Green qPCR SuperMix Kit (TransGen Biotech) according to the manufacturer’s instructions. Briefly, the qPCR reaction mixtures, consisting of 10 μL of SYBR Premix, 0.5 μL of PCR Forward Primer (10 μM), 0.5 μL of PCR Reverse Primer (10 μM), 1 μL of DNA template as well as 8 μL of H_2_O, were incubated for 30 cycles on the ABI 7900 RT–PCR system after pre-denaturation for 5 min at 95 °C. Each cycle contained denaturation for 10 s at 95 °C, annealing and extension for 30 s at 60 °C. The relative expression of all target genes comparing to glyceraldehyde-3-phosphate dehydrogenase (GAPDH) was determined by using the comparative Ct method. The primers were synthesized as shown in Supplementary Table 1.

### Western blot

Total proteins were extracted from breast cancer cells using RIPA lysis buffer and subsequently quantified using the Bradford method (Bio–Rad Laboratories, Hercules, CA, USA). Twenty micrograms of protein were loaded and separated by polyacrylamide gel electrophoresis and then transferred to PVDF membranes (Bio–Rad). After blocking with 5% BSA, membranes were incubated with rabbit anti-NFIL3 (1:1000, Cat.#11,773–1-AP, Proteintech, Rosemont, IL, USA), rabbit anti-NFKBIA (1:1000, Cat.#10,268–1-AP, Proteintech), mouse anti-GAPDH (1:5000, Cat.#600,04–1-Ig, Proteintech) or rabbit anti-tubulin (1:5000, Cat.#66,031–1-Ig, Proteintech) at 4 °C overnight. Then, membranes were incubated with peroxidase-conjugated goat anti-rabbit or goat anti-mouse secondary antibody for 1 h after washing. Finally, proteins were visualized through an enhanced chemiluminescence detection system (Thermo Scientific, Rockford, IL, USA). The relative gray value was measured using Image J software.

### Immunohistochemistry staining

Antigen retrieval treatment was performed in citrate buffer in a microwave over medium heat for 5 mins and then cooled to room temperature. After washing with PBS buffer three times, the sections were sealed by using goat serum (ZSGB Bio, Beijing, China) for 30 mins and incubated with a primary antibody against NFIL3 (1:250, Proteintech), NFKBIA (1:200, Proteintech), CD68 (1:400, Abcam Cat#ab213363, MA, USA), CD16 (1:200, Abcam Cat#ab183354), CD163 (1:500, Abcam Cat#ab182422) or CD8 (1:100, Abcam Cat#ab101500) at 4 °C overnight. After incubation with HRP-conjugated secondary antibody solution (PV-6001, ZSGB Bio) at room temperature for 30 mins, DAB (ZSGB Bio) was used for staining. The evaluation of immunohistochemistry staining included the percentage of positive tumor cells or normal glandular epithelial cells and localization of staining [16]. The expression of NFIL3 was localized in the nucleus, and NFKBIA was localized in the cytoplasm.

### Cell proliferation assay

Breast cancer cells were plated in 6-well plates at a density of 1 × 10^5^ cells/well. At 24, 48, and 72 h, cells were collected and resuspended in PBS. The cell number was counted by an automated cell counter (Countstar, Shanghai RuiYu, Biotech, CO. Ltd., Shanghai, China). All experiments were repeated three times.

### Clone formation assay

A total of 1000 cells per well were plated in 6-well plates and allowed to grow for approximately 15 days until the clones were visible to the naked eye. After washing with PBS, cell culture plates containing colonies were fixed with 3.7% formaldehyde for 10 mins and then stained with 0.2% crystal violet for 20 mins at room temperature. The number of clones was counted using Image J software.

### Invasion and migration assay

Cell invasion and migration assays were determined by using Transwell chambers (6.5 mm insert, 24-well plate, 8.0 μm polycarbonate membrane, BD Bioscience, Sparks, MD, USA) with or without Matrigel. Briefly, 4 × 10^4^ or 2 × 10^4^ cells/200 μL of breast cancer cell suspension was prepared separately in serum-free DMEM medium and plated in the upper chamber. Complete medium containing 10% FBS DMEM was added to the lower chamber. After culturing for 24 h, the upper chamber cells were wiped off using cotton swabs, and the chamber was fixed with 75% ethanol and stained with crystal violet. The cells were observed with a Leica microscopic imaging system. Ten fields were randomly selected for photography and statistical analysis.

A wound healing assay was also used to assess the migration of breast cancer cells. Breast cancer cells were seeded in 6-well plates and cultured until confluent. A straight line was scratched using a 200 μL pipettor spear head in each well. The 6-well plate was washed twice with PBS buffer to remove floating cells. The cells were cultured with serum-free DMEM medium. At 24, 48, and 72 h, scratch wound healing was recorded using an inverted microscope, and the scratch width was measured by Image J software for statistical analysis.

### Immunofluorescence assay

A total of 1 × 10^4^ cells were plated on coverslips and incubated at 37 °C for 48 h. Cells were fixed with 3.7% paraformaldehyde for 20 mins, permeabilized in 0.3% Triton X-100 for 10 mins, washed and blocked with 1% bovine serum albumin, and the coverslips were incubated with primary NF-κB antibody (1:100, Cat.#14,220–1-AP, Proteintech) at 4 °C overnight. Staining was achieved with secondary antibodies for 1 h at 37 °C. The slides were covered with coverslips and glycerin and viewed under a fluorescence microscope. Each test was repeated in triplicate.

### Dual-luciferase reporter assay

Hs578T cells were seeded in 24-well plates and grown to 40% confluence 24 h before transfection. Cells were cotransfected with luciferase constructs or empty pGL3 vector and Renilla luciferase plasmid (internal control) with or without NFIL3 overexpression plasmids. After 24 h, luciferase activities were assessed using the Dual-Luciferase Reporter Assay System according to the manufacturer’s instructions (Promega, Madison, WI, USA). The relative activity was calculated by the ratio of firefly luciferase activity to Renilla luciferase activity, and the fold-change of each reporter luciferase was normalized to the control. Data from three independent experiments are shown as the mean ± SD.

### Chromatin immunoprecipitation (ChIP) assay

ChIP assays were performed following the ChIP kit manufacturer’s protocol (Millipore Corporation, Billerica, MA, USA). Briefly, 1 × 10^7^ breast cancer cells were cultured, crosslinking was performed with 1% formalin (Sigma–Aldrich), the cells were lysed with SDS buffer, and sonication was used to fragment the DNA. An anti-NFIL3 antibody (Proteintech) was used to precipitate DNA-protein complexes, and normal isotype-matched IgG was used as a negative control. The DNA was extracted for PCR amplification. The sequences of NFIL3 and NFKBIA PCR primers are shown in Supplementary Table 1.

### Cycloheximide chase assay

NCM460, HCT15, MCF10A and Hs578T cells were seeded in 6-well plates at 60% confluency and cultured for 24 h. Then the cells were incubated with 100 μg/mL of cycloheximide (CHX). At 0, 3, 5 and 7 h after CHX addition, the cells were harvested and lysed on ice in RIPA buffer. The supernatant was subjected to SDS-PAGE and western blotting.

### Animal experiments

Four-week-old female Balb/c athymic nude mice (weight 12-15 g) were purchased from Vital River Laboratory (Beijing, China). All animal care and experimental procedures were approved by the Institutional Animal Care and Use committee of Harbin Medical University. A subcutaneous tumor-bearing experiment was used to assess the proliferation of tumor cells. There were five mice in each group, and a total of 2 × 10^6^ Hs578T cells were injected subcutaneously into each nude mouse. After the mice were sacrificed, the tumor weight and volume were measured. A lung metastasis mouse model generated by tail vein injection of breast cancer cells was used to evaluate cell metastasis, and there were ten mice in each group. Briefly, 4 × 10^6^ Hs578T cells were injected into the tail vein of each nude mouse. After the mice were sacrificed, the lungs were paraffin-embedded, and routine HE staining was performed. Intrapulmonary metastases were observed with a Leica imaging system and photographed. To investigate the effect of aspirin on the promotional roles of NFIL3 in growth and metastasis of Hs578T cells in vivo, the mice bearing tumors prepared as mentioned above were randomly divided into two groups and treated with PBS and Aspirin (100 mg/kg) respectively by intragastric administration every day until slaughtered at the endpoint of the experiments. Each group contained five mice for tumor growth assay and ten mice for metastasis assay respectively.

### Microarray hybridization

Total RNA from triplicate aliquots of Hs578T-control and Hs578T-shNFIL3 cells was extracted using TRIzol reagent as previously described. RNA quantity and quality were measured with a NanoDrop spectrophotometer (Thermo Fisher). RNA integrity was assessed by standard denaturing agarose gel electrophoresis. Sample preparation and microarray hybridization were performed based on Arraystar’s manufacturer’s protocols. Briefly, total RNA was reverse-transcribed into cDNA, synthesized into complementary RNA (cRNA) and labeled with Cyanine 3-CTP. The labeled cRNA was hybridized onto the microarray (Arraystar Inc., Shanghai, China). After washing, the arrays were scanned using an Agilent microarray scanner G2505C. The R software limma package was used for data normalization and calculation. The fold change between the control and shNFIL3 groups was calculated, and statistical significance was calculated by t-test.

### Statistical analysis

Data are expressed as the mean ± standard deviation. The data were tested by Student’s t-test or ANOVA using GraphPad Prism Software (GraphPad, San Diego, CA, USA). *p < 0.05, ** *p* < 0.01, *** *p* < 0.001 was considered statistically significant.

## Results

### Decreased NFIL3 mRNA indicates elevated NFIL3 protein in TNBC by screening the aberrant expression of rhythm genes in breast cancer

**Fig. 1:**
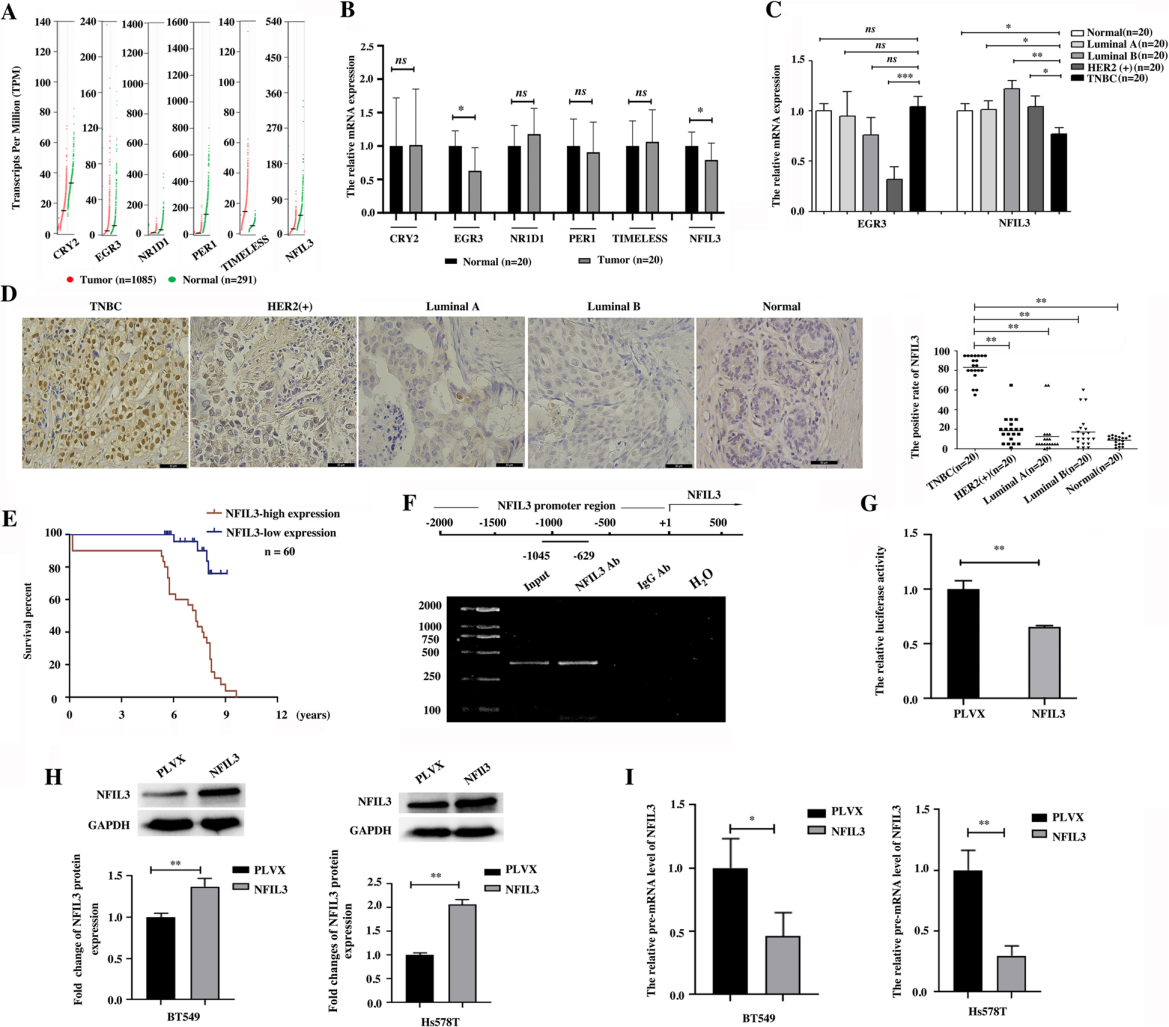
Decreased NFIL3 mRNA indicates elevated NFIL3 protein in TNBC. **A**. The expression of six rhythm genes is significantly changed in breast cancer compared with normal breast tissues based on the GEPIA database. **B**. The mRNA expression of six rhythm genes was compared using qRT–PCR in 20 normal breast tissues and 20 breast cancer tissues (*p < 0.05, ns means there was no significant difference). **C**. The mRNA expression of EGR3 and NFIL3 was compared in different subtypes of breast cancer by using qRT–PCR (*p < 0.05, **p < 0.01, ***p < 0.001, ns means there was no significant difference). **D**. The expression of NFIL3 protein in different subtypes of breast cancer was evaluated using IHC (magnification 400×, scale bars = 50 μm, **p < 0.01). **E**. High NFIL3 protein levels predict poor prognosis in TNBC patients. **F**. The binding of NFIL3 in the promoter region of the *NFIL3* gene was validated in Hs578T cells using a ChIP assay. **G**. A dual luciferase reporter assay was performed to test the suppressive effect of NFIL3 on the transcription of luciferase driven by the *NFIL3* gene promoter in Hs578T cells (**p < 0.01). This experiment was repeated in triplicate. **H**. The overexpression of NFIL3 protein in two TNBC cell lines was verified using western blot after forced expression of exogenous NFIL3. The relative expression of NFIL3 to GAPDH was normalized using Image J software (**p < 0.01). **I**. Relative NFIL3 pre-mRNA levels were detected by qRT–PCR after overexpressing exogenous NFIL3 (*p < 0.05, **p < 0.01)

We first investigated the aberrant expression of 32 rhythm genes in breast cancer based on the TCGA dataset and GTEx dataset by searching the GEPIA database. The expression of six rhythm genes, CRY2, NFIL3, PER1, EGR3, NR1D1 and TIMELESS, was significantly changed in breast cancer compared with normal breast tissues (Fig. 1A & Supplementary Fig. 1A). We further compared the mRNA levels of these six rhythm genes between 20 normal breast tissues and 20 breast cancer tissues by using qPCR and confirmed that the aberrant expression of NFIL3 and EGR3 in breast cancer is consistent with the results from the GEPIA database (Fig. 1B). When considering the expression patterns of NFIL3 and EGR3 in different subtypes of breast cancer, we found that EGR3 mRNA was significantly downregulated in the HER2-positive subtype of breast cancer, whereas NFIL3 mRNA was specifically lower in the TNBC subtype than in the other subtypes of breast cancer (Fig. 1C). We next focused on NFIL3 and evaluated the expression of NFIL3 protein between TNBC and other subtypes of breast cancer by using IHC. Surprisingly, we found that the expression of NFIL3 protein was significantly higher in TNBC than in other subtypes of breast cancer (Fig. 1D), which was contrary to the mRNA levels of the NFIL3 expression pattern in TNBC (Fig. 1C). Moreover, we demonstrated that high expression of NFIL3 protein is associated with poor prognosis of TNBC patients (Fig. 1E).

By investigating the expression change of NFIL3 mRNA across cancers, we found that NFIL3 was downregulated in most (13 out of 15) cancer types with significant NFIL3 alterations (Supplementary Fig. 1B). However, it is frequently reported that the expression of NFIL3 is elevated in various cancer types [17, 18]. To confirm the contrary results regarding the aberrant expression of NFIL3 at the mRNA and protein levels in cancer, we detected the expression of NFIL3 at the protein level in four additional cancer types with NFIL3 mRNA downregulation, including LUSC, LUAD, CESC and OV, by performing IHC. Our results showed that NFIL3 protein was significantly overexpressed in these detected cancer types compared with their normal tissues (Supplementary Fig. 1C), suggesting an opposite result regarding the expression of NFIL3 at the mRNA and protein levels in pancancer.

To determine the opposite results regarding NFIL3 expression at both the mRNA and protein levels, we first compared the degradation rate of NFIL3 between breast epithelial cell MCF10A and breast cancer cell Hs578T and between colon epithelial cell NCM460 and colonrectal cancer cell HCT15 respectively, and showed that there is no significant difference for degradation rate between cancer cell lines and their corresponding epithelial cells (Supplementary Fig. 1D-E). Then we analyzed the transcription factors that potentially bind the promoter region of the *NFIL3* gene by searching the Cistrome database. Interestingly, we found that NFIL3 potentially regulates its own transcription (Supplementary Fig. 1F), and several NFIL3 binding peaks within the promoter region of the *NFIL3* gene were identified (Supplementary Fig. 1G). Furthermore, we employed ChIP assays (Fig. 1F) and dual-luciferase reporter assays (Fig. 1G) to demonstrate that NFIL3 could bind the promoter region of the *NFIL3* gene and suppress its transcription. These results indicate that NFIL3 is a repressor of the transcription of the *NFIL3* gene, which may explain the opposite change in NFIL3 at the mRNA and protein levels. To test this hypothesis, we exogenously overexpressed NFIL3 driven by CMV enhancer in two TNBC cell lines (Fig. 1H) and an additional seven cancer cell lines representing six cancer types (Supplementary Fig. 1H-I) and detected the impact of elevated NFIL3 protein on the transcription of the endogenous *NFIL3* gene. We showed that pre-mRNA of NFIL3 was significantly downregulated upon increase of exogenous NFIL3 protein in all detected cancer cell lines (Fig. 1I & Supplementary Fig. 1 J). These results suggest that NFIL3 is a transcriptional repressor of NFIL3 transcription, and decreased NFIL3 mRNA indicates elevated NFIL3 protein levels across cancers.

### NFIL3 protein promotes the proliferation and metastasis of TNBC cells

**Fig. 2:**
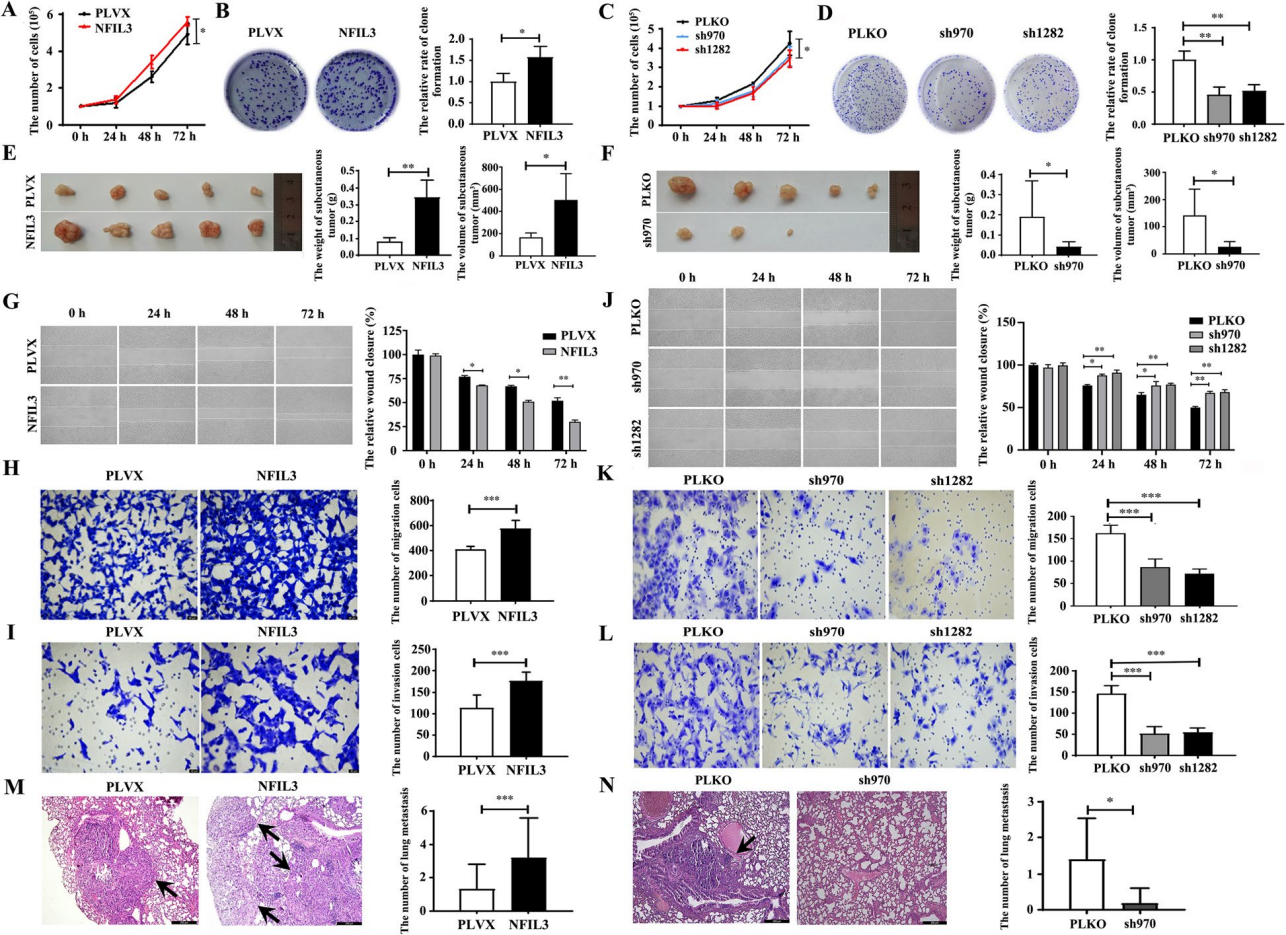
NFIL3 promotes the proliferation and metastasis of TNBC cells in vitro and in vivo. **A**. Overexpression of NFIL3 promoted the proliferation of Hs578T cells, as determined by the cell counting test (*p < 0.05). **B**. Overexpression of NFIL3 promoted the clone formation rate of Hs578T cells (*p < 0.05). **C**. Knockdown of NFIL3 inhibited the proliferation of Hs578T cells, as determined by the cell counting test (*p < 0.05). **D**. Knockdown of NFIL3 inhibits the clone formation rate of Hs578T cells (**p < 0.01). **E**. NFIL3 overexpression promoted the growth of Hs578T cells in a xenograft mouse model, as determined by evaluating the weight and volume of tumors (*p < 0.05, **p < 0.01). **F**. Knockdown of NFIL3 inhibited the growth of Hs578T cells in a xenograft mouse model by evaluating the weight and volume of tumors (*p < 0.05). **G**. Overexpression of NFIL3 promotes the wound healing of Hs578T cells (*p < 0.05, **p < 0.01). **H**. Overexpression of NFIL3 enhances the migration of Hs578T cells, as detected by Transwell assay (magnification, 200×; ***p < 0.001). **I**. Overexpression of NFIL3 enhances the invasion of Hs578T cells, as detected by Transwell assay (magnification, 200×; ***p < 0.001). **J**. Knockdown of NFIL3 inhibits the wound healing of Hs578T cells (*p < 0.05, **p < 0.01). **K**. Knockdown of NFIL3 inhibited the migration of Hs578T cells, as detected by Transwell assay (magnification, 200×; ***p < 0.001). **L**. Knockdown of NFIL3 inhibited the invasion of Hs578T cells, as detected by Transwell assay (magnification, 200×; ***p < 0.001). **M**. Overexpression of NFIL3 promotes lung metastasis of Hs578T cells using a tail vein injection model (magnification, 100×; scale bars = 200 μm; the black arrow indicates the metastatic foci; ***p < 0.001). **N**. Knockdown of NFIL3 suppresses lung metastasis of Hs578T cells using a tail vein injection model (magnification, 100×; scale bars = 200 μm; the black arrow indicates the metastatic foci; *p < 0.05)

To detect the potential roles of NFIL3 protein in TNBC progression, we investigated the effects of NFIL3 protein on the proliferation and metastasis of two TNBC cell lines, BT549 and Hs578T, by gain-of-function and loss-of-function strategies, respectively. The stable overexpression of exogenous NFIL3 in two TNBC cell lines at both the mRNA and protein levels was confirmed by qRT–PCR and western blot assays (Supplementary Fig. 2A, B). Cell counting assays and clone formation assays revealed that increasing NFIL3 protein promoted the proliferation of Hs578T cells (Fig. 2A-B) and BT549 cells (Supplementary Fig. 2C, D), whereas knockdown of endogenous NFIL3 (Supplementary Fig. 2E, F) inhibited the proliferation of Hs578T cells (Fig. 2C, D) and BT549 cells (Supplementary Fig. 2G, H) in vitro. Consistently, the subcutaneous tumor formation assay also demonstrated that elevating NFIL3 protein promoted the growth of Hs578T cells (Fig. 2E), whereas decreasing NFIL3 protein inhibited the growth of Hs578T cells in vivo (Fig. 2F). Using wound healing assays and Transwell assays, we demonstrated that overexpression of NFIL3 protein promoted the migration and invasion of Hs578T cells (Fig. 2G-I) and BT549 cells (Supplementary Fig. 2I, J), whereas decrease of NFIL3 protein inhibited the migration and invasion of both cell lines (Fig. 2J-L and Supplementary Fig. 2 K, L). Consistently, the mouse tail vein injection model also revealed that elevated NFIL3 protein promoted the lung metastasis of Hs578T cells (Fig. 2M), whereas decrease of NFIL3 protein inhibited the lung metastasis of Hs578T cells (Fig. 2N). These results suggest that NFIL3 protein promotes the progression of TNBC cells in vitro and in vivo.

### NFIL3 regulates tumor associated inflammation in breast cancer

**Fig. 3:**
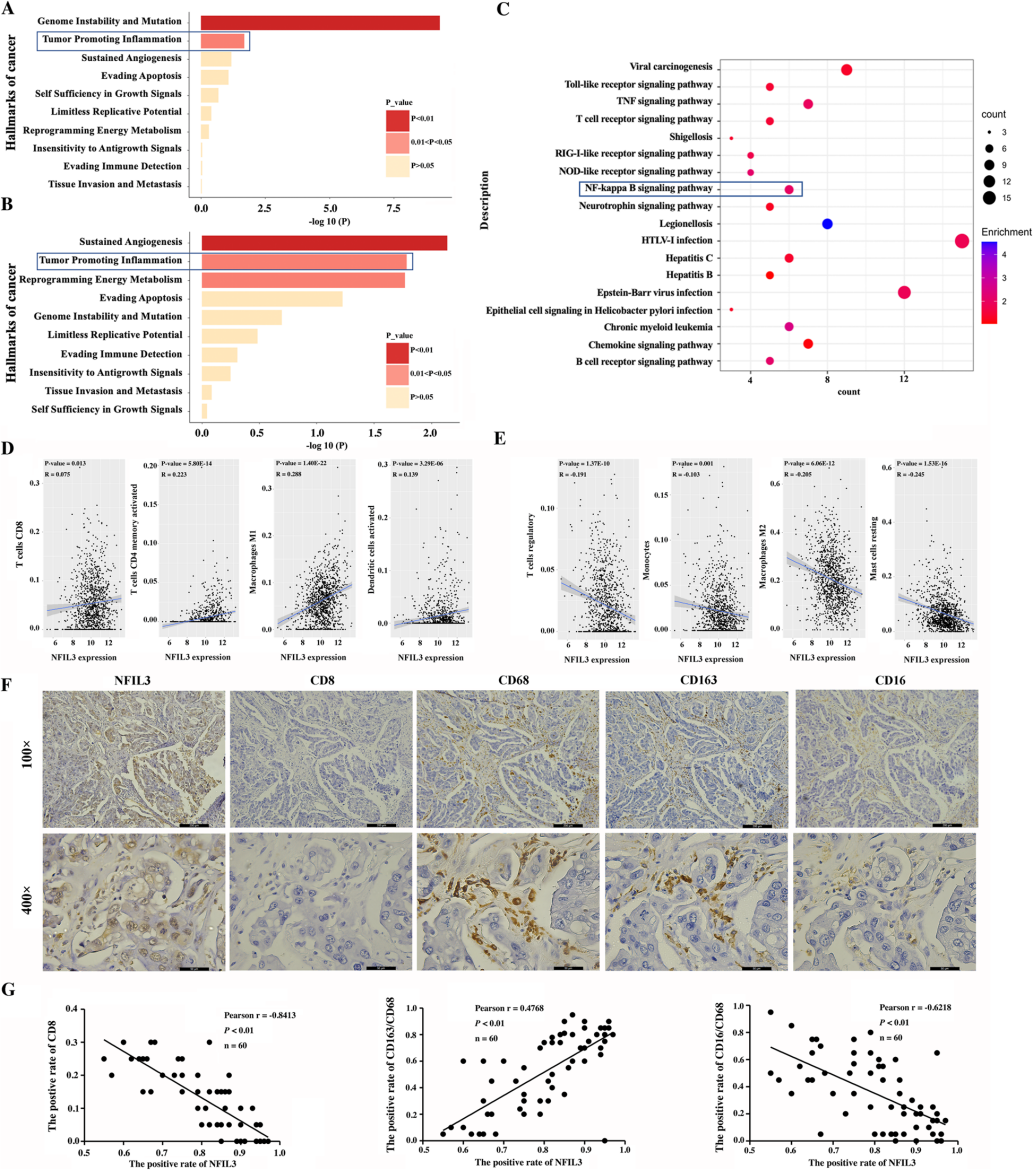
NFIL3 regulates cancer associated inflammation in breast cancer. **A**. Functional enrichment analysis of downregulated genes associated with 10 hallmarks of cancer upon knocking down NFIL3. **B**. Functional enrichment analysis of upregulated genes associated with 10 hallmarks of cancer upon knocking down NFIL3. **C**. Enrichment analysis of significantly changed genes associated with inflammatory pathways. The size of dots represents the number of genes in a specific inflammatory pathway. The color of the dots represents the enrichment degree of genes with a specific inflammatory pathway. **D**. The positive correlation between NFIL3 mRNA level and the infiltration of CD8+ T cells, activated CD4 memory T cells, M1 cells and activated DCs in the breast cancer was obtained by Pearson Correlation analysis based on the TCGA dataset (*p* < 0.05). **E**. The negative correlation between NFIL3 mRNA level and the infiltration of resting mast cells, regulatory T (Treg) cells, monocytes and M2 cells in the breast cancer was obtained by Pearson Correlation analysis based on the TCGA dataset (*p* < 0.05). **F**. The representative expression of NFIL3, CD8, CD68, CD163 and CD16 at protein level was detected by IHC (magnification, 100×, scale bars = 200 μm; magnification, 400×, scale bars = 50 μm). **G**. Pearson correlation analysis between the expression of NFIL3protein and the level of infiltrated CD8+ T cells, M1 and M2 cells in 60 TNBC tissues (*p* < 0.01)

To investigate the molecular mechanism by which NFIL3 protein promotes the progression of TNBC cells, we detected global gene expression changes upon NFIL3 knockdown using a mRNA array when NFIL3 was considered a transcription factor. We identified 1990 genes that were significantly changed upon NFIL3 knockdown in Hs578T cells, including 891 upregulated genes and 1099 downregulated genes (Supplementary Fig. 3A, B & Supplementary Table 2). The analysis of functional enrichment in ten hallmarks of cancer showed that both downregulated and upregulated genes were significantly enriched in inflammation (Fig. 3A, B). Then, we analyzed the major inflammatory pathways affected by NFIL3 knockdown and showed that both innate immune signaling pathways (such as Toll-like receptor signaling, RIG-I-like receptor signaling and NOD-like receptor signaling) and adaptive immune signaling pathways (such as T cell receptor signaling and B cell receptor signaling) were significantly enriched (Fig. 3C). By analyzing the correlation between NFIL3 mRNA levels and the infiltration of 22 inflammatory cells in breast cancer tissues based on the TCGA dataset (Fig. 3D-E & Supplementary Fig. 3C, D), we showed that NFIL3 mRNA levels were positively correlated with the infiltration levels of type I macrophages (M1), CD4+ memory T cells, CD8+ T cells, dendritic cells (DCs), follicular T helper cells, M0 cells and neutrophils (Fig. 3D & Supplementary Fig. 3C), while negatively correlated with the levels of Treg cells, type II macrophages (M2), monocytes, resting mast cells, naïve CD4+ T cells, γδ T cells and eosinophils (Fig. 3E & Supplementary Fig. 3D), respectively. Furthermore, we detected the correlation between NFIL3 protein levels and the amount of infiltrated M1, M2 and CD8+ T cells in 60 TNBC tissues and confirmed that NFIL3 protein levels were positively correlated with infiltrated M2 cells whereas negatively correlated with infiltrated M1 cells and CD8 + T cells, respectively (Fig. 3F-G). These results suggest that NFIL3 regulates tumor associated inflammation in breast cancer.

### NFIL3 enhances the activity of NF-κB signaling by inhibiting the expression of NFKBIA in TNBC cells

**Fig. 4:**
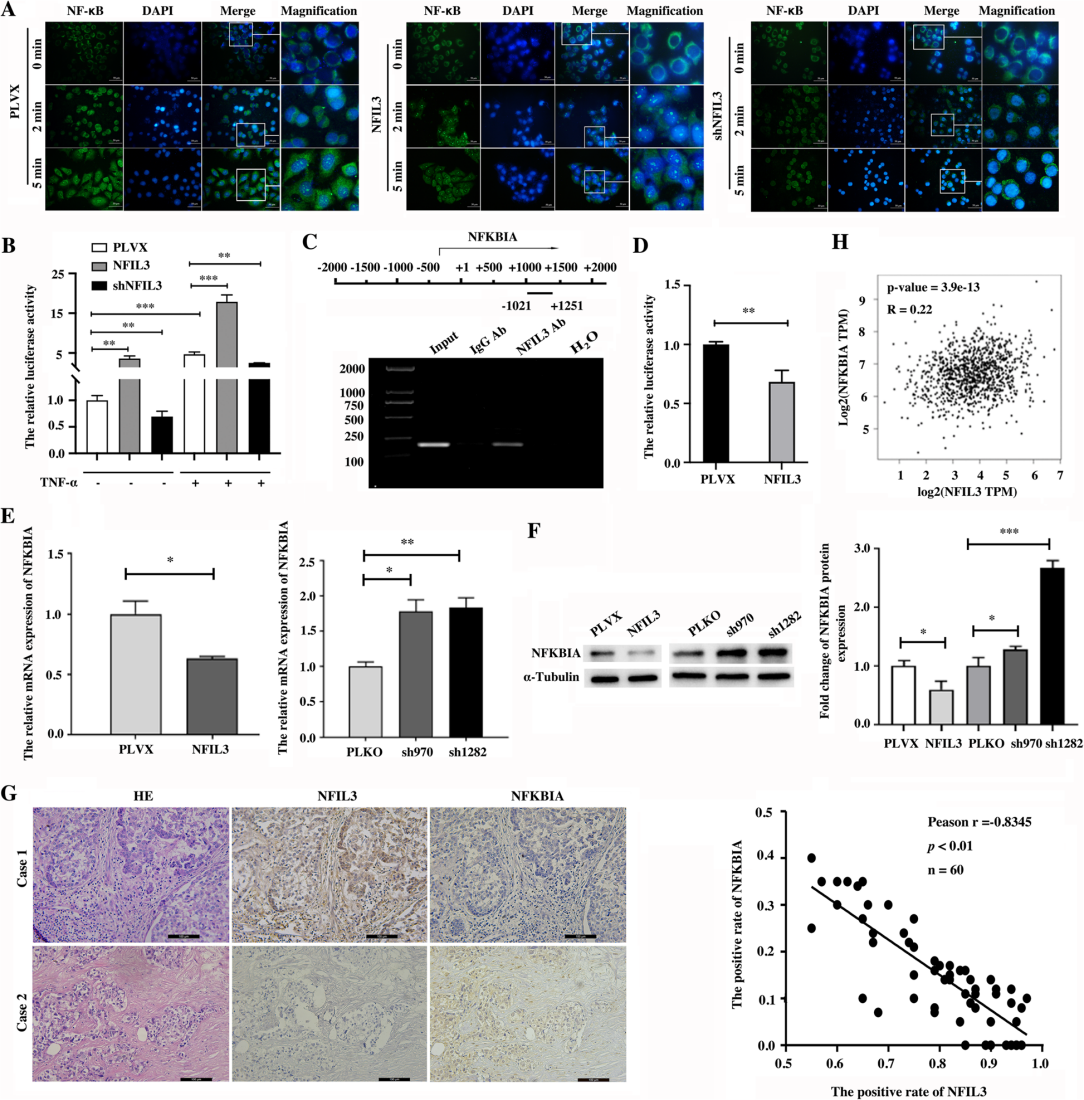
NFIL3 enhances the activity of NF-κB signaling and inhibits the transcription of NFKBIA in TNBC cells. **A**. Translocation of NF-κB from the cytoplasm to the nucleus was detected using immunofluorescence to indicate the activation of NF-κB signaling upon changing the expression of NFIL3 in Hs578T cells with TNF-α (25 ng/mL) stimulation (magnification 400×, scale bars = 50 μm). **B**. A luciferase reporter assay was used to detect the activity of NF-κB signaling in Hs578T cells upon NFIL3 overexpression or knockdown with or without TNF-α (25 ng/mL) stimulation (**p < 0.01, ***p < 0.001). The experiment was repeated in triplicate. **C**. The binding of NFIL3 in the promoter region of the *NFKBIA* gene was validated in Hs578T cells using a ChIP assay. **D**. A dual luciferase reporter assay was performed to test the suppressive effect of NFIL3 on the transcription of luciferase driven by the *NFKBIA* gene promoter in Hs578T cells (**p < 0.01). This experiment was repeated in triplicate. **E-F**. The expression change of NFKBIA at the mRNA and protein levels in Hs578T cells was detected using qPCR and western blots, respectively, upon NFIL3 overexpression or knockdown. (*p < 0.05, **p < 0.01, ***p < 0.001). **G**. Pearson correlation analysis between the expression of NFIL3 and NFKBIA in breast cancer tissues using IHC (magnification, 200×, scale bars = 100 μm, *p* < 0.01). **H**. Pearson correlation analysis between NFIL3 and NFKBIA in breast cancer tissues based on the TCGA dataset

The NF-κB signaling pathway, a well-known inflammatory pathway associated with cancer progression, was enriched in the top ten inflammatory signaling pathways regulated by NFIL3 (Fig. 3C). Next, we evaluated the roles of NFIL3 protein in regulating the activity of NF-κB signaling. Translocation of NF-κB from the cytoplasm into the nucleus is required and indicates the activation of NF-κB signaling. By performing an immunofluorescence assay, we detected the distribution changes of NF-κB in Hs578T cells stimulated with TNF-α upon altering the expression of NFIL3. After stimulation with TNF-α, NF-κB is quickly translocated from the cytoplasm to the nucleus, and the overexpression of NFIL3 protein significantly enhances the translocation of NF-κB into the nucleus at different time points, whereas decrease of NFIL3 protein blocks the nuclear translocation of NF-κB (Fig. 4A). Subsequently, we confirmed that overexpression of NFIL3 protein significantly enhances NF-κB activity, whereas knockdown of NFIL3 significantly inhibits NF-κB activity using a dual-luciferase reporter assay (Fig. 4B). These results demonstrated that NFIL3 effectively promotes NF-κB activation in TNBC cells.

In total, six NF-κB signaling pathway-related genes were significantly changed upon NFIL3 knockdown, three of which were downregulated, while three were upregulated (Supplementary Fig. 4A), and the expression change of these six genes upon NFIL3 knockdown was confirmed by qPCR (Supplementary Fig. 4B). Among the six NFIL3-regulated genes in the NF-κB signaling pathway, four (TRIM25, BCL10, RIPK1 and NFKBIA) are regulators of NF-κB activity, whereas the others (GADD45B and CXCL2) are downstream target genes of NF-κB (Supplementary Fig. 4A). The downregulation of BCL10 and RIPK1 and upregulation of NFKBIA may all contribute to suppressing the activity of NF-κB upon NFIL3 knockdown. Considering that NFIL3 is a transcriptional suppressor and NFKBIA, a canonical suppressor of NF-κB activation, is significantly upregulated in NFIL3 knockdown cells, we focused on NFKBIA for further validation as the direct target of NFIL3.

ChIP-sequencing data showed that there are several NFIL3 binding sites in the promoter region of the *NFKBIA* gene (Supplementary Fig. 4C). We employed ChIP assays and dual-luciferase reporter assays to demonstrate that NFIL3 binds the *NFKBIA* gene (Fig. 4C) and inhibits its transcription (Fig. 4D) in Hs578T cells. We further showed that NFKBIA was decreased in NFIL3-overexpressing cells but was increased in NFIL3 knockdown cells at both the mRNA and protein levels in both Hs578Tcells (Fig. 4E-F) and BT549 cells (Supplementary Fig. 4D-E). Moreover, we detected the protein levels of NFIL3 and NFKBIA in breast cancer tissues using IHC and revealed that the NFIL3 protein level was negatively correlated with the NFKBIA protein level in breast cancer tissues (Fig. 4G). A similar result was obtained in normal breast tissues (Supplementary Fig. 4F). Consistently, we analyzed the correlation between NFIL3 and NFKBIA in the TCGA dataset and found that NFIL3 mRNA was positively correlated with NFKBIA mRNA in both breast cancer (Fig. 4H) and pancancer (Supplementary Fig. 4G). These results show that NFKBIA is a direct target of NFIL3 protein and is transcriptionally repressed by NFIL3 in TNBC cells.

### NFKBIA suppresses the activity of NF-κB signaling and blocks the proliferation and metastasis of TNBC cells in vitro and in vivo

**Fig. 5:**
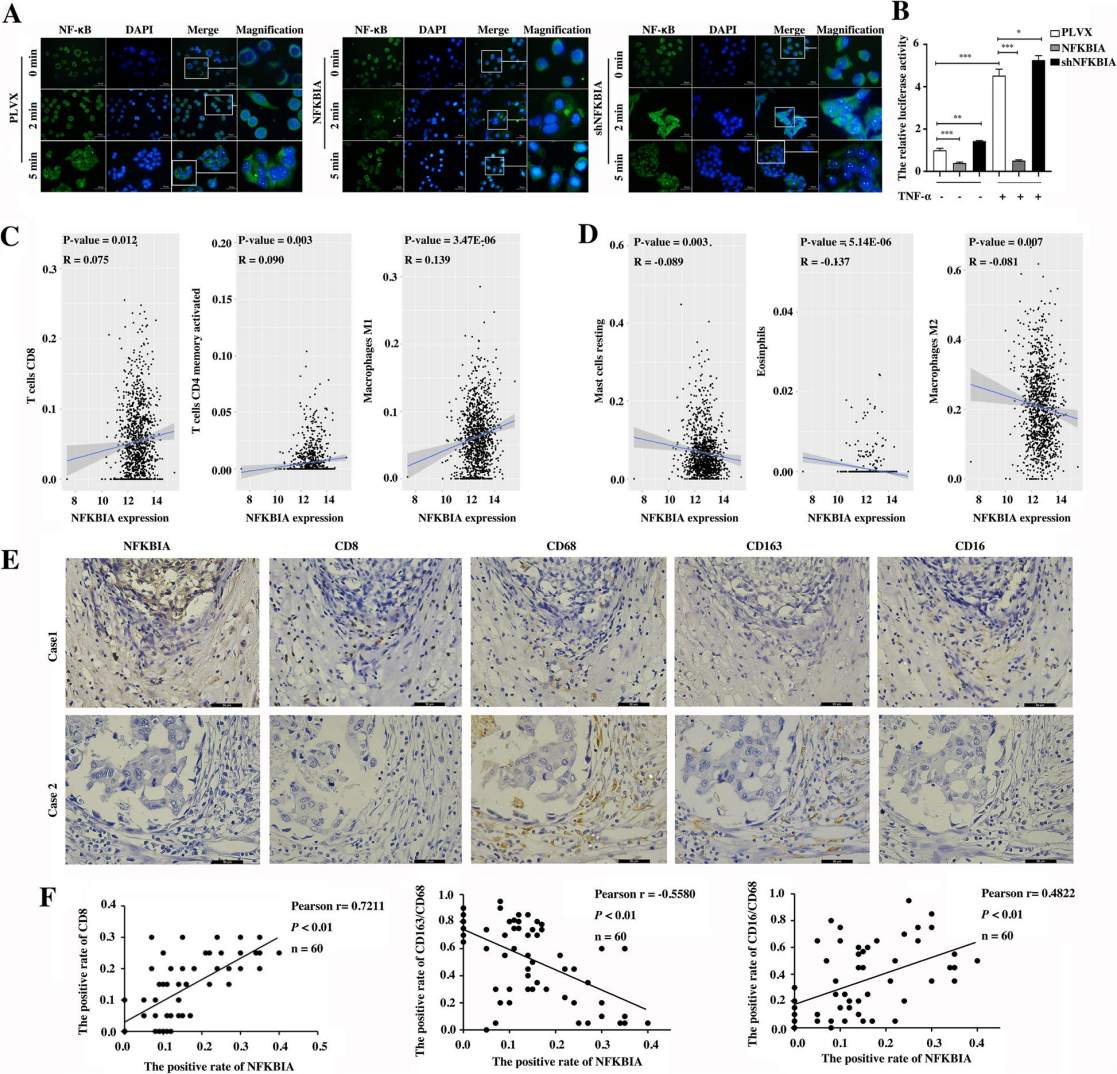
NFKBIA suppresses the activity of NF-κB signaling and is correlated with the infiltration of immune cells in breast cancer. **A**. The impact of NFKBIA overexpression or knockdown on the translocation of NF-κB from the cytoplasm to the nucleus in Hs578T cells detected by immunofluorescence assay. **B**. A luciferase reporter assay was used to detect the activity of NF-κB signaling in Hs578T cells upon NFKBIA overexpression or knockdown with or without TNF-α (25 ng/mL) stimulation (*p < 0.05, **p < 0.01, ***p < 0.001). The experiment was repeated in triplicate. **C**. The positive correlation between NFKBIA mRNA level and the infiltration of CD8+ T cells, activated CD4 memory T cells and M1 cells in the breast cancer was obtained by Pearson Correlation analysis based on the TCGA dataset (*p* < 0.05). **D**. The negative correlation between NFKBIA mRNA level and the infiltration of resting mast cells, eosinophils cells and M2 cells in the breast cancer was obtained by Pearson Correlation analysis based on the TCGA dataset (*p* < 0.05). **E**. The representative expression of NFKBIA, CD8, CD68, CD163 and CD16 at protein level was detected by IHC (magnification, 400×, scale bars = 50 μm). **F**. Pearson correlation analysis between the expression of NFKBIA protein and the level of infiltrated CD8+ T cells, M1 and M2 cells in 60 TNBC tissues (*p* < 0.01)

We examined the role of NFKBIA in the activity of NF-κB signaling and tumor associated inflammation in breast cancer. First, the stable overexpression (Supplementary Fig. 5A, B) and knockdown (Supplementary Fig. 5C-D) of NFKBIA at both the mRNA and protein levels in BT549 and Hs578T cells were confirmed by qRT–PCR and western blot assays, respectively. Then, we detected the effect of NFKBIA on NF-κB activity. As expected, NFKBIA knockdown enhanced the translocation of NF-κB from the cytoplasm into the nucleus, whereas overexpression of NFKBIA blocked the nuclear translocation of NF-κB in Hs578T cells (Fig. 5A). Consistently, we showed that knockdown of NFKBIA significantly enhances NF-κB activity, whereas overexpression of NFKBIA significantly inhibits NF-κB activity using a dual-luciferase reporter assay (Fig. 5B). Additionally, by analyzing the correlation between NFKBIA mRNA levels and the infiltration of 22 inflammatory cells in breast cancer tissues based on the TCGA dataset (Fig. 5C, D & Supplementary Fig. 5E-F), we showed that NFKBIA mRNA levels were positively correlated with infiltration levels of M1, CD4+ T cells, CD8+ T cells and naïve B cells (Fig. 5C & Supplementary Fig. 5E), whereas negatively correlated with infiltration levels of M2 cells, resting mast cells and eosinphils in breast cancer tissues based on the TCGA dataset (Fig. 5D). The correlation between NFKBIA protein levels and the amount of infiltrated M1, M2 and CD8+ T cells was also confirmed in breast cancer tissues using IHC assays (Fig. 5E, F). These results suggest that NFKBIA is a suppressor of NF-κB signaling and it regulates tumor associated inflammation in TNBC different from NFIL3.

**Fig. 6:**
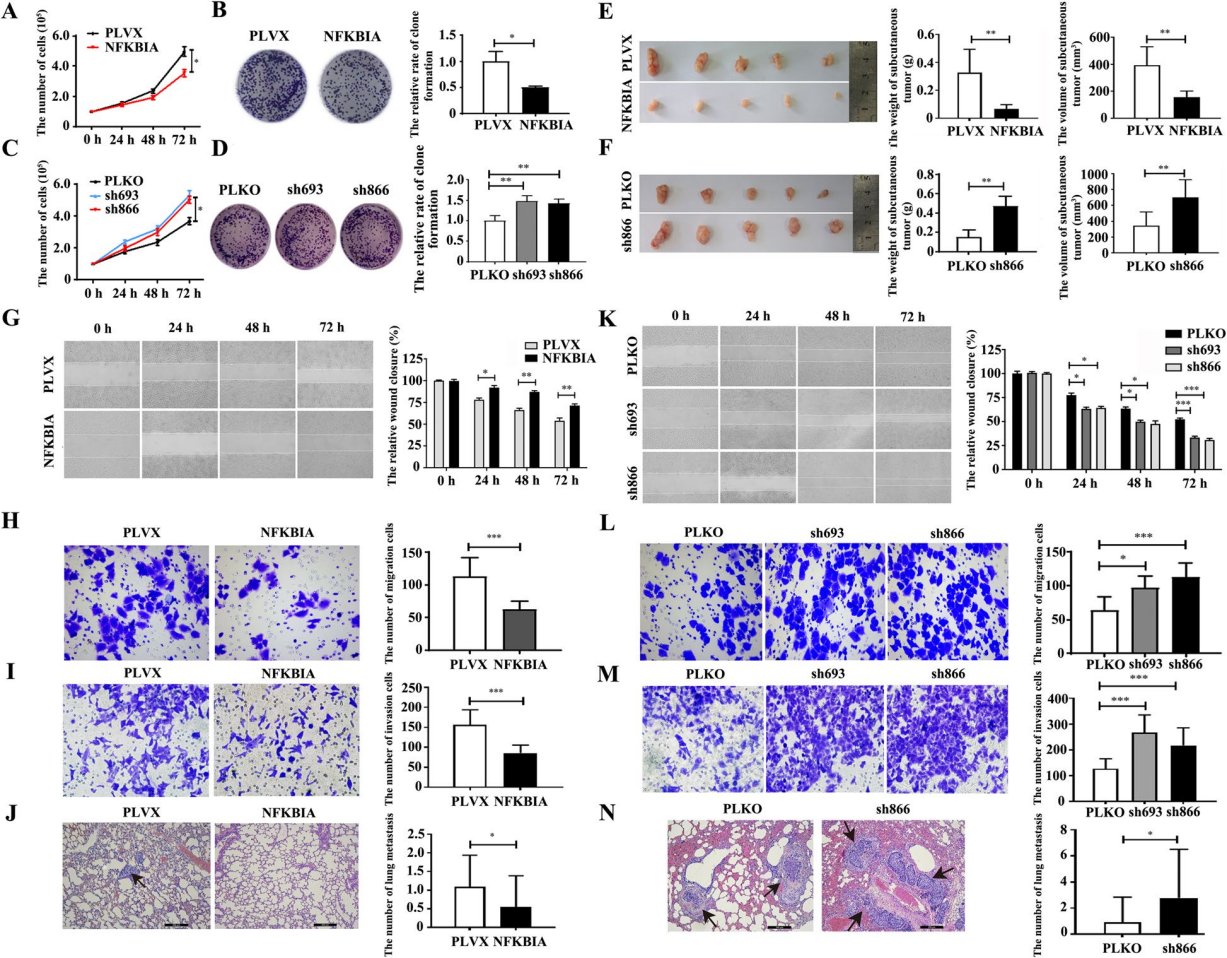
NFKBIA blocks the proliferation and metastasis of TNBC cells. **A**. Overexpression of NFKBIA inhibited the proliferation of Hs578T cells, as determined by the cell counting test (*p < 0.05). **B**. Overexpression of NFKBIA inhibits the clone formation rate of Hs578T cells (*p < 0.05). **C**. Knockdown of NFKBIA promoted the proliferation of Hs578T cells, as determined by the cell counting test (*p < 0.05). **D**. Knockdown of NFKBIA promotes the clone formation rate of Hs578T cells (**p < 0.01). **E**. NFKBIA overexpression inhibited the growth of Hs578T cells in a xenograft mouse model by evaluating the weight and volume of tumors (**p < 0.01). **F**. Knockdown of NFKBIA promotes the growth of Hs578T cells in a xenograft mouse model by evaluating the weight and volume of tumors (**p < 0.01). **G**. Overexpression of NFKBIA inhibits the wound healing of Hs578T cells (*p < 0.05, **p < 0.01). **H**. Overexpression of NFKBIA inhibited the migration of Hs578T cells, as detected by Transwell assay (magnification, 200×; ***p < 0.001). **I**. Overexpression of NFKBIA inhibited the invasion of Hs578T cells, as detected by Transwell assay (magnification, 200×; ***p < 0.001). **J**. Overexpression of NFKBIA inhibited lung metastasis of Hs578T cells using a tail vein injection model (magnification, 100×; scale bars = 200 μm; the black arrow indicates the metastatic foci; *p < 0.05). **K**. Knockdown of NFKBIA promotes the wound healing of Hs578T cells (*p < 0.05, ***p < 0.001). **L**. Knockdown of NFKBIA promoted the migration of Hs578T cells, as detected by Transwell assay (magnification, 200×; *p < 0.05, ***p < 0.001). **M**. Knockdown of NFKBIA promoted the invasion of Hs578T cells, as detected by Transwell assay (magnification, 200×; ***p < 0.001). **N**. Knockdown of NFKBIA promotes lung metastasis of Hs578T cells using a tail vein injection model (magnification, 100×; scale bars = 200 μm; the black arrow indicates the metastatic foci; *p < 0.05)

Next, using cell counting and clone formation assays, we found that overexpression of NFKBIA inhibited proliferation, whereas knockdown of NFKBIA promoted the proliferation of Hs578T cells (Fig. 6A-D) and BT549 cells (Supplementary Fig. 6A-D). Consistently, overexpression of NFKBIA inhibited the growth of subcutaneous Hs578T tumors (Fig. 6E), whereas knockdown of NFKBIA promoted the growth of subcutaneous Hs578T tumors in a nude mouse model (Fig. 6F). Furthermore, overexpression of NFKBIA not only inhibited the migration and invasion of Hs578T cells (Fig. 6G-I) and BT549 cells (Supplementary Fig. 6E, F) in vitro but also decreased the lung metastasis of Hs578T cells in a mouse tail vein injection model (Fig. 6J). Knockdown of NFKBIA promoted the migration and invasion of Hs578T cells (Fig. 6K-M) and BT549 cells (Supplementary Fig. 6G, H) in vitro and enhanced the metastasis of Hs578T cells in vivo (Fig. 6N). These results suggest that NFKBIA, unlike oncogenic NFIL3, is a tumor suppressor that inhibits the progression of breast cancer cells.

### NFIL3 promotes the activation of NF-κB and the progression of breast cancer by inhibiting NFKBIA

**Fig. 7:**
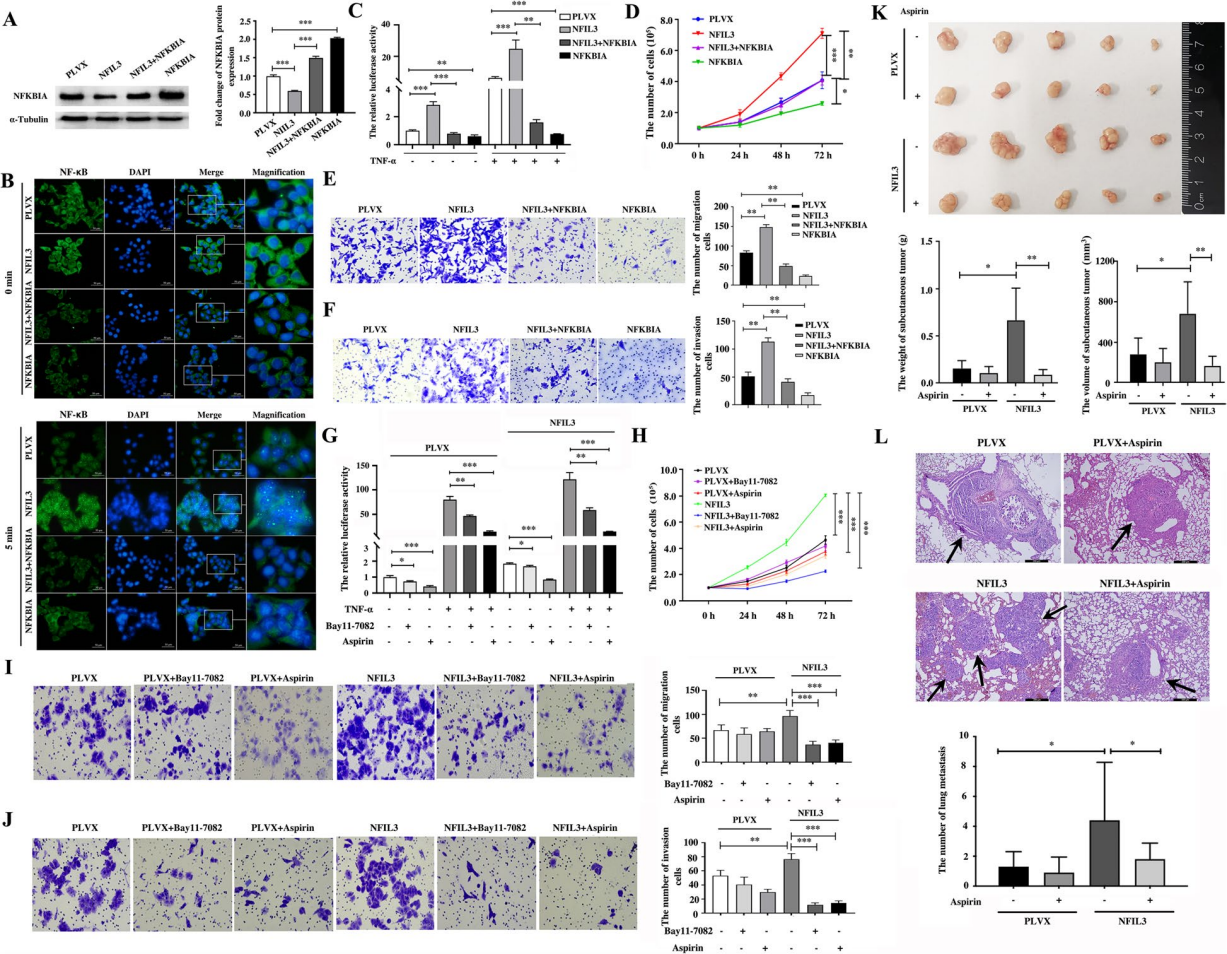
NFIL3 promotes the proliferation, migration and invasion of TNBC cells by activating the NF-κB inflammatory pathway through inhibiting NFKBIA. **A**. The restored expression of NFKBIA in Hs578T cells with NFIL3 overexpression was confirmed by western blot. The relative NFKBIA expression was analyzed using Image J software (***p < 0.001). **B**. Translocation of NF-κB detected using an immunofluorescence assay was employed to investigate the effect of NFKBIA restoration on the activation of NF-κB signaling in Hs578T cells with NFIL3 overexpression. (magnification, 400×, scale bars = 50 μm.) **C**. A luciferase reporter assay was used to detect the effect of NFKBIA restoration on the activity of NF-κB signaling in Hs578T cells upon NFIL3 overexpression with or without TNF-α (25 ng/mL) stimulation (**p < 0.01, ***p < 0.001). The experiment was repeated in triplicate. **D**. The effect of NFKBIA restoration on the proliferation of Hs578T cells with NFIL3 overexpression (*p < 0.05, **p < 0.01, ***p < 0.001). **E-F**. The effects of NFKBIA restoration on the migration and invasion of Hs578T cells with NFIL3 overexpression (**p < 0.01). **G**. Aspirin (4 mmol/L) and Bay11–7082 (10 μmol/L) blocked the activation of NF-κB signaling triggered by NFIL3 overexpression or TNF-α (25 ng/mL) stimulation (*p < 0.05, **p < 0.01, ***p < 0.001). **H**. Both aspirin (4 mmol/L) and Bay11–7082 (10 μmol/L) significantly inhibited the promoting effect of NFIL3 on the proliferation of Hs578T cells (***p < 0.001). **I-J**. Both aspirin (4 mmol/L) and Bay11–7082 (10 μmol/L) significantly inhibited the promotional effect of NFIL3 on the migration and invasion of Hs578T cells (**p < 0.01, ***p < 0.001). **K**. Aspirin suppressed the promotional roles of NFIL3 in promoting the growth of Hs578T cells in a xenograft mouse model by evaluating the weight and volume of tumors (*p < 0.05, **p < 0.01). **L**. Aspirin suppressed the promotional roles of NFIL3 in promoting the lung metastasis of Hs578T cells using a tail vein injection mouse model (magnification, 100×; scale bars = 200 μm; the black arrow indicates the metastatic foci; *p < 0.05)

To further evaluate the critical roles of NFKBIA in mediating the effects of NFIL3 on the activation of NF-κB and the progression of TNBC, we simultaneously overexpressed both NFKBIA and NFIL3 in Hs578T (Fig. 7A) and BT549 cells (Supplementary Fig. 7A) and detected the impact of both NFKBIA and NFIL3 overexpression on the activity of NF-κB and the proliferation, migration and invasion of both breast cancer cell lines. We found that rescuing the expression of NFKBIA in breast cancer cells with NFIL3 overexpression blocked the rapid translocation of NF-κB from the cytoplasm into the nucleus triggered by NFIL3 overexpression in Hs578T cells (Fig. 7B). Consistently, we confirmed that rescuing the expression of NFKBIA inhibited the promotional effect of NFIL3 on NF-κB activity using a dual-luciferase reporter assay (Fig. 7C). We further demonstrated that rescuing the expression of NFKBIA partially reversed the promotional effect of NFIL3 on the proliferation of Hs578T cells (Fig. 7D) and BT549 cells (Supplementary Fig. 7B) and on the migration and invasion of Hs578T (Fig. 7E, F) and BT549 cells (Supplementary Fig. 7C, D). Taken together, we showed that NFIL3 promotes the progression of TNBC by suppressing NFKBIA and then enhancing the activity of the NF-κB signaling pathway.

### Anti-inflammatory strategies targeting NF-κB activity block the oncogenic role of NFIL3 protein in TNBC

Because activating NF-κB activity by inhibiting the expression of NFKBIA mimics the roles of NFIL3 in the progression of TNBC, we proposed that anti-inflammatory strategies targeting NF-κB activity may block the oncogenic role of NFIL3 protein in TNBC. To test this hypothesis, we evaluated the effects of aspirin and Bay11–7082, two inhibitors of NF-κB activity that inhibit the phosphorylation of IKKB and IKKA, respectively [19, 20], on the roles of NFIL3 protein in stimulating NF-κB activity and promoting the progression of TNBC. As expected, both aspirin and Bay11–7082 not only significantly blocked the activation of NF-κB signaling mediated by NFIL3 protein, as detected by dual-luciferase reporter assay (Fig. 7G), but also significantly inhibited the promotional effect of NFIL3 on the proliferation of Hs578T cells (Fig. 7H) and BT549 cells (Supplementary Fig. 7E) and on the migration and invasion of Hs578T (Fig. 7I, J) and BT549 cells in vitro (Supplementary Fig. 7F, G). Moreover, aspirin dramatically suppressed the promotional roles of NFIL3 in the growth (Fig. 7K) and metastasis (Fig. 7L) of Hs578T cells in nude mice. Taken together, we showed that strategies targeting NF-κB activity block the oncogenic roles of NFIL3 protein in TNBC.

## Discussion

In this study, we revealed that the expression of NFIL3 at the mRNA and protein levels was not coincident across cancers with an unclear mechanism. The rhythm gene NFIL3 is a transcriptional repressor that downregulates the expression of its target genes [21] . For the first time, we demonstrated that NFIL3 protein suppressed the transcription of *NFIL3* gene, which explained the paradoxical expression pattern of NFIL3 at the mRNA and protein levels across cancers. The overall downregulation of NFIL3 at the mRNA level in pancancer indicates the upregulation of NFIL3 at the protein level. However, the mechanisms underlying the elevation of NFIL3 protein across cancers are unknown and deserve further study in the future. Additionally, our previous research found that some rhythm genes other than NFIL3 regulate their own transcription, including BHLHE40, EGR3, CLOCK, and CRY1 [10].

We demonstrated that NFIL3 promotes the progression of TNBC. To the best of our knowledge, this is the first study on the functions of the rhythm gene *NFIL3* in TNBC. This result is consistent with previous observations regarding the function of NFIL3 in other cancer types. Elevated expression of NFIL3 promoted the invasion and migration of lung cancer cells [22]. NFIL3 also promoted thyroid cancer proliferation [23] and induced EMT, cell migration and experimental metastasis of colorectal cancer cells [24].

Numerous studies have shown that NFIL3 regulates inflammatory responses. For example, NFIL3 is necessary for the development of NK cells and type 1 innate lymphoid cells (ILC1s), and it plays a critical role in the development of other mucosal tissue-associated innate lymphocytes. Therefore, NFIL3 is crucial for intestinal innate immune defense against acute bacterial infection [25]. Additionally, NFIL3 suppresses human inflammatory bowel diseases (IBD) by repressing the transcription of IL-12p40, which mediates T cell inflammatory responses to the enteric microbiota [26].

Consistent with these findings, we also identified that NFIL3-regulated genes were enriched in inflammatory signaling pathways. Especially, we demonstrated that NFIL3 positively regulates the activity of NF-κB. Elevated activation of NF-κB signaling promotes the proliferation, migration and invasion of cancer cells and induces angiogenesis in various cancer types [27, 28].

It has been reported that TRIM25, BCL10 and RIPK1 positively regulate the activation of NF-κB [29–31], whereas NFKBIA suppresses the activation of NF-κB [32]. In this study, we revealed that NFIL3 negatively regulates the expression of NFKBIA but positively regulates the expression of BCL10 and RIPK1, consistent with the roles of NFIL3 in regulating NF-κB activity. However, we singled out NFKBIA for further investigation in this study for two reasons. First, the expression change of NFKBIA upon NFIL3 alteration is consistent with the function of NFIL3 as a transcriptional suppressor. Second, NFKBIA is directly upstream of NF-κB, and it binds NF-κB in the cytoplasm and inhibits the translocation of NF-κB into the nucleus [33]. Mutations, gene polymorphisms and haploid karyotypes of NFKBIA in various malignant tumors result in elevated NF-κB signaling activity and contribute to promoting the proliferation and metastasis of cancer cells [34, 35]. Our results demonstrated that inhibiting NFKBIA is the major mechanism by which NFIL3 promotes the progression of TNBC. However, we could not exclude that NFIL3 may also activate NF-κB signaling by enhancing the expression of NF-κB activators, such as BCL10 and RIPK1, although the molecular mechanisms by which NFIL3 increases their expression are unclear.

## Conclusion

In summary, NFIL3 protein is elevated in TNBCs compared with both normal breast tissues and other subtypes of breast cancer, and promotes the progression of TNBC cells by activating the NF-κB signaling pathway through suppressing the expression of NFKBIA. Anti-inflammatory strategies targeting NF-κB activity block the oncogenic roles of NFIL3 in TNBC. This study may provide a new target for TNBC prevention and therapy.

## Supplementary Information

**Additional file 1: Figure S1.** Decreased NFIL3 mRNA indicates elevated NFIL3 protein in pancancer. A. The expression of 26 rhythm genes was not altered in breast cancer compared with normal breast tissues. B. NFIL3 is significantly downregulated at the mRNA level in most cancer types (*p < 0.05). C. The expression of NFIL3 protein in four representative cancer types was evaluated using IHC (magnification 200×, scale bars = 100 μm; *p < 0.05, **p < 0.01). D. MCF10A and Hs578T cells were treated with CHX for 0 h, 3 h, 5 h and 7 h, then cells were harvested and the protein level of NFIL3 was detected by western blot, and the relative expression of NFIL3 to GAPDH was normalized using Image J software (*p < 0.05, **p < 0.01, ***p < 0.001, ns means not significant). E. NCM460 and HCT15 cells were treated with CHX for 0 h, 3 h, 5 h and 7 h, then cells were harvested and the protein level of NFIL3 was detected by western blot, and the relative expression of NFIL3 to GAPDH was normalized using Image J software (*p < 0.05, **p < 0.01, ns means not significant). F. Top 20 transcriptional regulators potentially regulate the transcription of the human NFIL3 gene identified using Cistrome DB Toolkit. Regulatory potential (RP) is a score to estimate how possible the factor can regulate a gene. G. There are several NFIL3 binding sites in the promoter region of the NFIL3 gene revealed by ChIP sequencing in the Cistrome database. The binding site highlighted with a box was selected for further validation in colon cancer cell lines. H. The overexpression of NFIL3 protein in seven cancer cell lines representing six cancer types was verified by western blot after forced expression of exogenous NFIL3. I. The relative protein expression of NFIL3 to GAPDH was normalized using Image J software (*p < 0.05, **p < 0.01, ***p < 0.001). J. The relative NFIL3 pre-mRNA level was detected by qRT–PCR after overexpressing exogenous NFIL3 (*p < 0.05, **p < 0.01, ***p < 0.001).

**Additional file 2: Figure S2.** NFIL3 promotes the proliferation, migration and invasion of BT549 cells. A. Overexpression of NFIL3 in Hs578T cells and BT549 cells was confirmed using qRT–PCR (**p < 0.01). B. Overexpression of NFIL3 in Hs578T cells and BT549 cells was confirmed by western blot (***p < 0.001). C. Overexpression of NFIL3 promoted the proliferation of BT549 cells, as determined by the cell counting test (*p < 0.05). D. Overexpression of NFIL3 promoted the clone formation rate of BT549 cells (*p < 0.05). E. Knockdown of NFIL3 in Hs578T cells and BT549 cells was confirmed using qRT–PCR (**p < 0.01). F. Knockdown of NFIL3 in Hs578T cells and BT549 cells was confirmed by western blot (***p < 0.001). G. Knockdown of NFIL3 inhibited the proliferation of BT549 cells, as determined by the cell counting test (**p < 0.01). H. Knockdown of NFIL3 inhibited the clone formation rate of BT549 cells (**p < 0.01). I. Overexpression of NFIL3 enhanced the migration of BT549 cells, as detected by Transwell assay (magnification, 200×; ***p < 0.001). J. Overexpression of NFIL3 enhanced the invasion of BT549 cells, as detected by Transwell assay (magnification, 200×; ***p < 0.001). K. Knockdown of NFIL3 inhibited the migration of BT549 cells, as detected by Transwell assay (magnification, 200×; ***p < 0.001). L. Knockdown of NFIL3 inhibited the invasion of BT549 cells, as detected by Transwell assay (magnification, 200×; ***p < 0.001).

**Additional file 3: Figure S3.** NFIL3 inhibits the expression of NFKBIA. A. Numbers of significantly altered genes in Hs578T cells upon NFIL3 knockdown. B. Heatmap of significantly altered genes in Hs578T cells upon NFIL3 knockdown. C-D. The significant correlation between NFIL3 mRNA level and the infiltration of resting CD4 memory T cells, naïve CD4 T cells, follicular T helper cells, γδ T cells, M0 cells, neutrophils and eosinophils in the breast cancer was obtained by Pearson Correlation analysis based on the TCGA dataset (*p* < 0.05). E. There is no significant correlation between NFIL3 mRNA level and the infiltration of other types of immune cells in breast cancer detected by Pearson Correlation analysis based on the TCGA dataset (*p* > 0.05).

**Additional file 4: Figure S4.** NFIL3 inhibits the transcription of NFKBIA. A. Diagram of the NF-κB signaling pathway based on the KEGG database. Red represents upregulated genes, and green represents downregulated genes upon NFIL3 knockdown. B. The mRNA expression change of six NF-κB signaling pathway-related genes was confirmed by qRT–PCR (*p < 0.05, **p < 0.01). C. There are several binding sites of NFIL3 in the promoter region of the *NFKBIA* gene based on ChIP-sequencing data from the Cistrome database. D-E. Changes in NFKBIA expression at the mRNA and protein levels in BT549 cells upon NFIL3 overexpression or knockdown were detected using qRT–PCR and western blot, respectively (*p < 0.05, **p < 0.01). F. Pearson correlation analysis between the expression of NFIL3 and NFKBIA in normal breast epithelial tissues using IHC (magnification, 200×, scale bars = 100 μm, *p* < 0.01). G. Pearson correlation analysis between NFIL3 and NFKBIA in pancancer based on the TCGA dataset (r = 0.23).

**Additional file 5: Figure S5.** Establishment of TNBC cell lines with stable NFKBIA overexpression or knock down and the correlation analysis between NFKBIA mRNA level and infiltration of immune cells in breast cancer. A. Overexpression of NFKBIA in Hs578T cells and BT549 cells was confirmed using qRT–PCR (***p < 0.001). B. Overexpression of NFKBIA in Hs578T cells and BT549 cells was confirmed by western blot (***p < 0.001). C. Knockdown of NFKBIA in BT549 cells and Hs578T cells was confirmed by qRT–PCR (***p < 0.001). D. Knockdown of NFKBIA in BT549 cells and Hs578T cells was confirmed by western blot (**p < 0.01, ***p < 0.001). E. The positive correlation between NFKBIA mRNA level and the infiltration of resting CD4 memory T cells and naïve B cells in the breast cancer was obtained by Pearson Correlation analysis based on the TCGA dataset (*p* < 0.05). F. There is no significant correlation between NFKBIA mRNA level and the infiltration of other types of immune cells in breast cancer detected by Pearson Correlation analysis based on the TCGA dataset (*p* > 0.05).

**Additional file 6: Figure S6.** NFKBIA suppresses the proliferation, migration and invasion of BT549 cells. A. Overexpression of NFKBIA inhibited the proliferation of BT549 cells, as determined by the cell counting test (**p < 0.01). B. Overexpression of NFKBIA inhibited the clone formation rate of BT549 cells (*p < 0.05). C. Knockdown of NFKBIA promoted the proliferation of BT549 cells, as determined by the cell counting test (***p < 0.001). D. Knockdown of NFKBIA promoted the clone formation rate of BT549 cells (**p < 0.01). E. Overexpression of NFKBIA inhibited the migration of BT549 cells, as detected by Transwell assay (magnification, 200×; ***p < 0.001). F. Overexpression of NFKBIA inhibited the invasion of BT549 cells, as detected by Transwell assay (magnification, 200×; ***p < 0.001). G. Knockdown of NFKBIA promoted the migration of BT549 cells, as detected by Transwell assay (magnification, 200×; ***p < 0.001). H. Knockdown of NFKBIA promoted the invasion of BT549 cells, as detected by Transwell assay (magnification, 200×; ***p < 0.001).

**Additional file 7: Figure S7.** NFIL3 promotes the proliferation, migration and invasion of BT549 cells by promoting the NF-κB inflammatory pathway through inhibiting NFKBIA. A. The restored expression of NFKBIA in BT549 cells with NFIL3 overexpression was confirmed by western blot. The relative NFKBIA expression was analyzed using Image J software (**p < 0.01, ***p < 0.001). B. The effect of NFKBIA restoration on the proliferation of BT549 cells with NFIL3 overexpression (*p < 0.05, **p < 0.01, ***p < 0.001). C-D. The effects of NFKBIA restoration on the migration and invasion of BT549 cells overexpressing NFIL3 (**p < 0.01). E. Both aspirin (4 mmol/L) and Bay11–7082 (10 μmol/L) significantly inhibited the promoting effect of NFIL3 on the proliferation of BT549 cells (***p < 0.001). F-G. Both aspirin (4 mmol/L) and Bay11–7082 (10 μmol/L) significantly inhibited the promotional effect of NFIL3 on the migration and invasion of BT549 cells (*p < 0.05, ***p < 0.001).

**Additional file 8: Supplementary Table 1.**

**Additional file 9: Supplementary Table 2.**

## Data Availability

The datasets used and analyzed during the current study are available from the corresponding author on reasonable request.

## Abbreviations

NFIL3: nuclear factor interleukin 3 regulated
TNBC: Triple-negative breast cancer
TCGA: The Cancer Genome Atlas
ChIP: Chromatin Immunoprecipitation
cRNA: Complementary RNA
DCs: Dendritic cells
M1: type I macrophages
M2: type II macrophages
IBD: Inflammatory bowel disease
