# Supplementary figures and images for: Elevated expression of the rhythm gene NFIL3 promotes the progression of TNBC by activating NF-κB signaling through suppression of NFKBIA transcription
Source: J Exp Clin Cancer Res. 2022 Feb 18;41:67. doi: 10.1186/s13046-022-02260-1 (PMC8855542; doi:10.1186/s13046-022-02260-1)

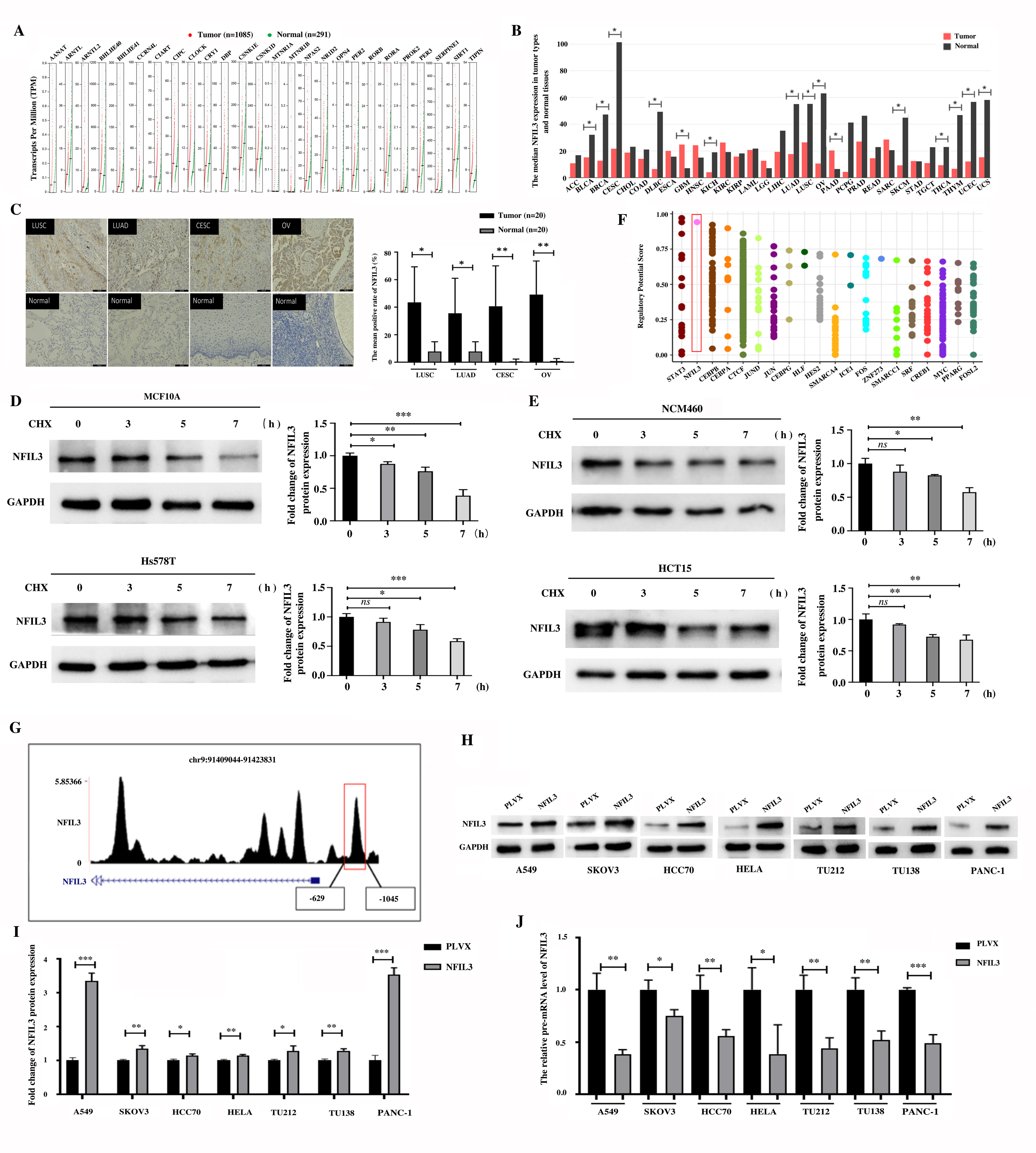

Supplement: Supplementary file 1 — Additional file 1: Figure S1. Decreased NFIL3 mRNA indicates elevated NFIL3 protein in pancancer. A. The expression of 26 rhythm genes was not altered in breast cancer compared with normal breast tissues. B. NFIL3 is significantly downregulated at the mRNA level in most cancer types (*p < 0.05). C. The expression of NFIL3 protein in four representative cancer types was evaluated using IHC (magnification 200×, scale bars = 100 μm; *p < 0.05, **p < 0.01). D. MCF10A and Hs578T cells were treated with CHX for 0 h, 3 h, 5 h and 7 h, then cells were harvested and the protein level of NFIL3 was detected by western blot, and the relative expression of NFIL3 to GAPDH was normalized using Image J software (*p < 0.05, **p < 0.01, ***p < 0.001, ns means not significant). E. NCM460 and HCT15 cells were treated with CHX for 0 h, 3 h, 5 h and 7 h, then cells were harvested and the protein level of NFIL3 was detected by western blot, and the relative expression of NFIL3 to GAPDH was normalized using Image J software (*p < 0.05, **p < 0.01, ns means not significant). F. Top 20 transcriptional regulators potentially regulate the transcription of the human NFIL3 gene identified using Cistrome DB Toolkit. Regulatory potential (RP) is a score to estimate how possible the factor can regulate a gene. G. There are several NFIL3 binding sites in the promoter region of the NFIL3 gene revealed by ChIP sequencing in the Cistrome database. The binding site highlighted with a box was selected for further validation in colon cancer cell lines. H. The overexpression of NFIL3 protein in seven cancer cell lines representing six cancer types was verified by western blot after forced expression of exogenous NFIL3. I. The relative protein expression of NFIL3 to GAPDH was normalized using Image J software (*p < 0.05, **p < 0.01, ***p < 0.001). J. The relative NFIL3 pre-mRNA level was detected by qRT–PCR after overexpressing exogenous NFIL3 (*p < 0.05, **p < 0.01, ***p < 0.001). [file 13046_2022_2260_MOESM1_ESM.tif]

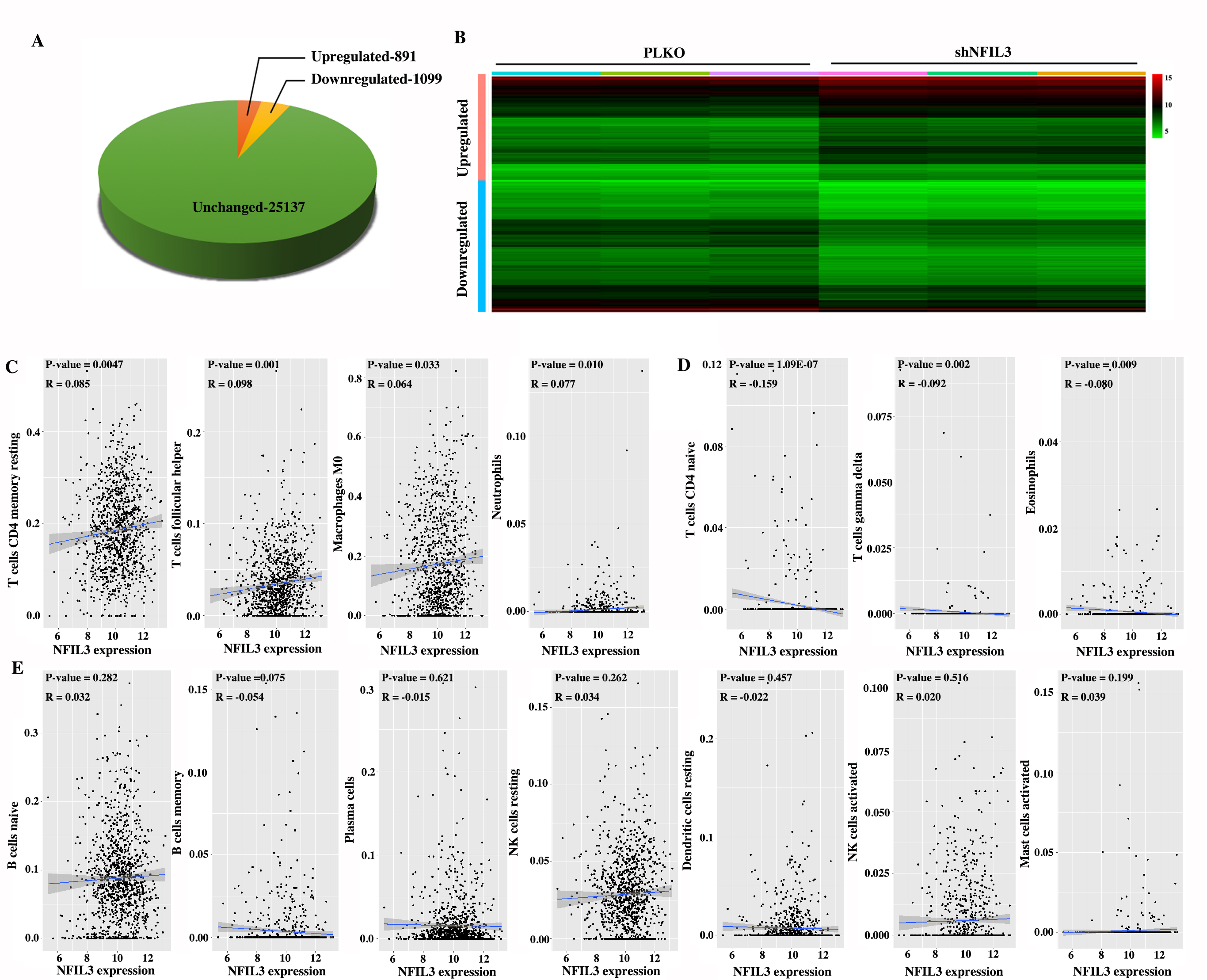

Supplement: Supplementary file 3 — Additional file 3: Figure S3. NFIL3 inhibits the expression of NFKBIA. A. Numbers of significantly altered genes in Hs578T cells upon NFIL3 knockdown. B. Heatmap of significantly altered genes in Hs578T cells upon NFIL3 knockdown. C-D. The significant correlation between NFIL3 mRNA level and the infiltration of resting CD4 memory T cells, naïve CD4 T cells, follicular T helper cells, γδ T cells, M0 cells, neutrophils and eosinophils in the breast cancer was obtained by Pearson Correlation analysis based on the TCGA dataset (p < 0.05). E. There is no significant correlation between NFIL3 mRNA level and the infiltration of other types of immune cells in breast cancer detected by Pearson Correlation analysis based on the TCGA dataset (p > 0.05). [file 13046_2022_2260_MOESM3_ESM.tif]

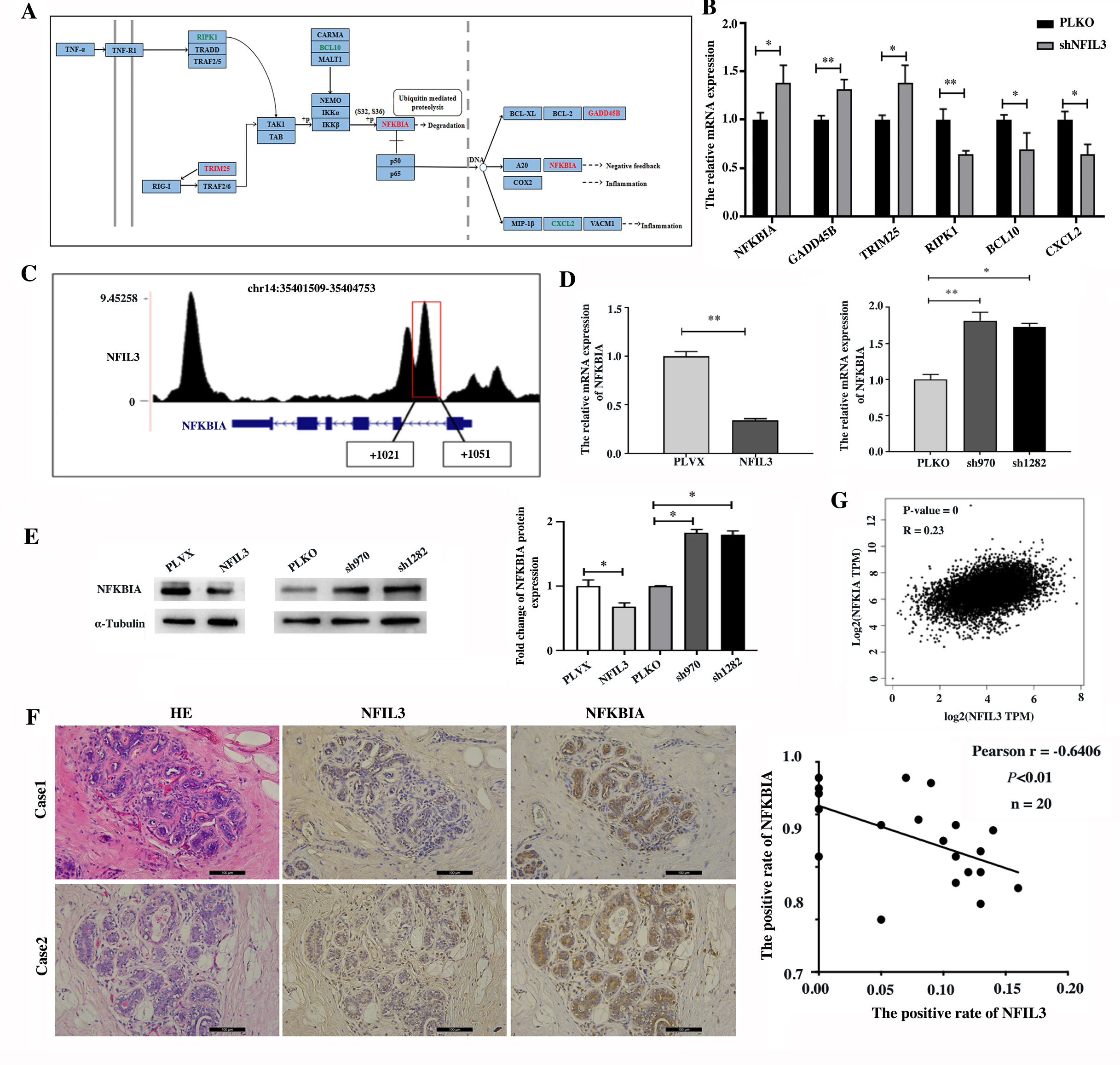

Supplement: Supplementary file 4 — Additional file 4: Figure S4. NFIL3 inhibits the transcription of NFKBIA. A. Diagram of the NF-κB signaling pathway based on the KEGG database. Red represents upregulated genes, and green represents downregulated genes upon NFIL3 knockdown. B. The mRNA expression change of six NF-κB signaling pathway-related genes was confirmed by qRT–PCR (*p < 0.05, **p < 0.01). C. There are several binding sites of NFIL3 in the promoter region of the NFKBIA gene based on ChIP-sequencing data from the Cistrome database. D-E. Changes in NFKBIA expression at the mRNA and protein levels in BT549 cells upon NFIL3 overexpression or knockdown were detected using qRT–PCR and western blot, respectively (*p < 0.05, **p < 0.01). F. Pearson correlation analysis between the expression of NFIL3 and NFKBIA in normal breast epithelial tissues using IHC (magnification, 200×, scale bars = 100 μm, p < 0.01). G. Pearson correlation analysis between NFIL3 and NFKBIA in pancancer based on the TCGA dataset (r = 0.23). [file 13046_2022_2260_MOESM4_ESM.tif]

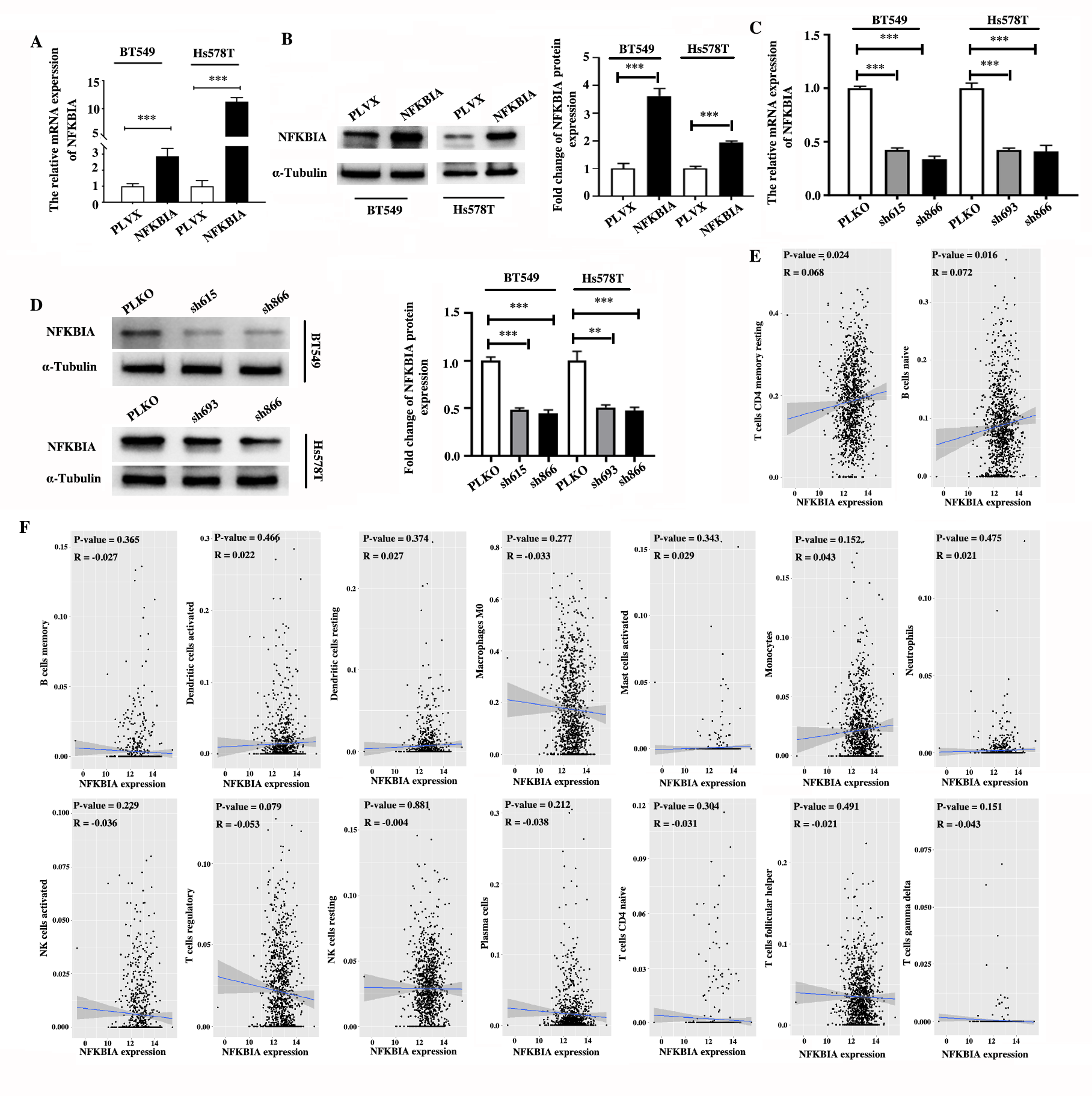

Supplement: Supplementary file 5 — Additional file 5: Figure S5. Establishment of TNBC cell lines with stable NFKBIA overexpression or knock down and the correlation analysis between NFKBIA mRNA level and infiltration of immune cells in breast cancer. A. Overexpression of NFKBIA in Hs578T cells and BT549 cells was confirmed using qRT–PCR (***p < 0.001). B. Overexpression of NFKBIA in Hs578T cells and BT549 cells was confirmed by western blot (***p < 0.001). C. Knockdown of NFKBIA in BT549 cells and Hs578T cells was confirmed by qRT–PCR (***p < 0.001). D. Knockdown of NFKBIA in BT549 cells and Hs578T cells was confirmed by western blot (**p < 0.01, ***p < 0.001). E. The positive correlation between NFKBIA mRNA level and the infiltration of resting CD4 memory T cells and naïve B cells in the breast cancer was obtained by Pearson Correlation analysis based on the TCGA dataset (p < 0.05). F. There is no significant correlation between NFKBIA mRNA level and the infiltration of other types of immune cells in breast cancer detected by Pearson Correlation analysis based on the TCGA dataset (p > 0.05). [file 13046_2022_2260_MOESM5_ESM.tif]

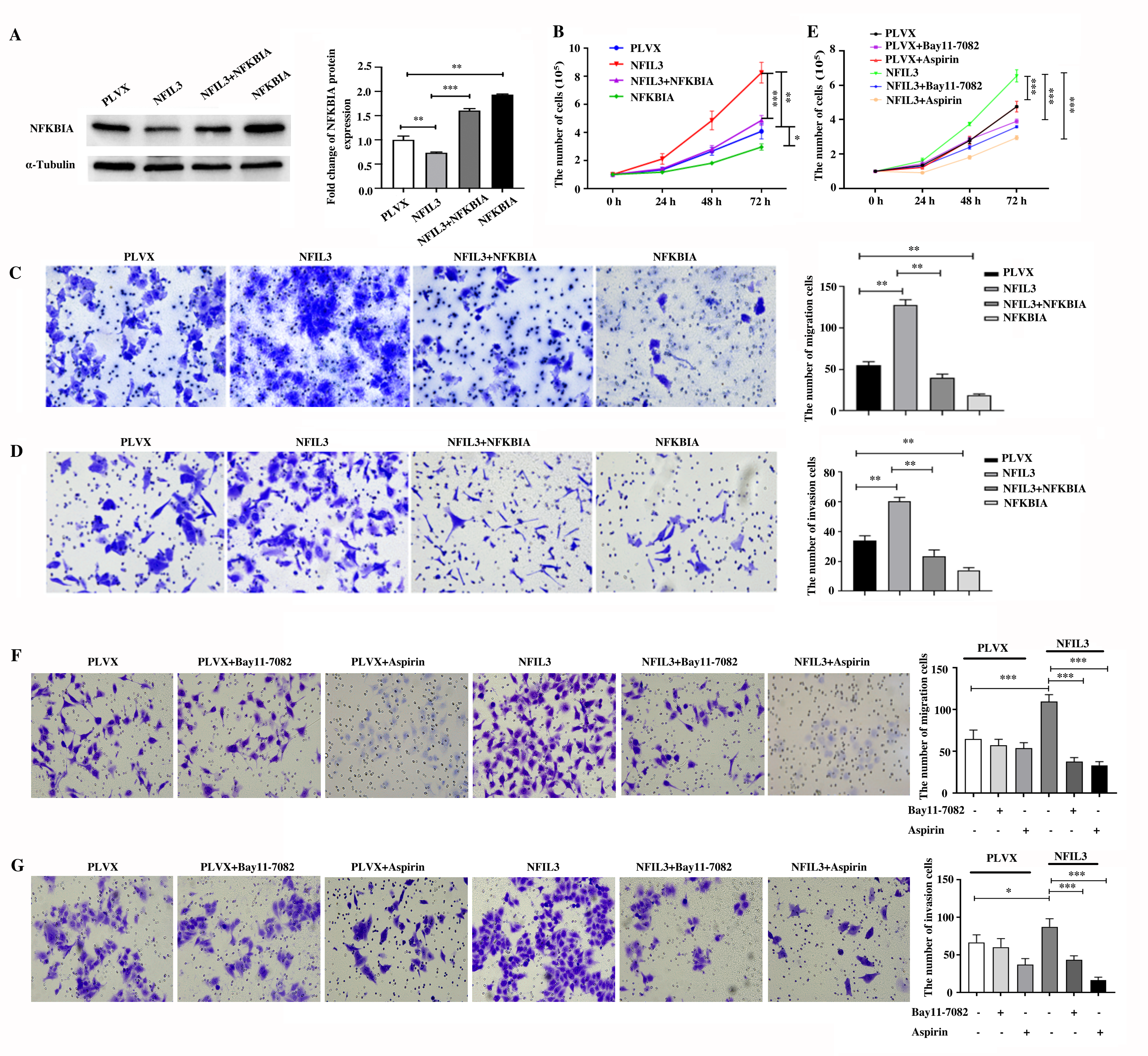

Supplement: Supplementary file 7 — Additional file 7: Figure S7. NFIL3 promotes the proliferation, migration and invasion of BT549 cells by promoting the NF-κB inflammatory pathway through inhibiting NFKBIA. A. The restored expression of NFKBIA in BT549 cells with NFIL3 overexpression was confirmed by western blot. The relative NFKBIA expression was analyzed using Image J software (**p < 0.01, ***p < 0.001). B. The effect of NFKBIA restoration on the proliferation of BT549 cells with NFIL3 overexpression (*p < 0.05, **p < 0.01, ***p < 0.001). C-D. The effects of NFKBIA restoration on the migration and invasion of BT549 cells overexpressing NFIL3 (**p < 0.01). E. Both aspirin (4 mmol/L) and Bay11–7082 (10 μmol/L) significantly inhibited the promoting effect of NFIL3 on the proliferation of BT549 cells (***p < 0.001). F-G. Both aspirin (4 mmol/L) and Bay11–7082 (10 μmol/L) significantly inhibited the promotional effect of NFIL3 on the migration and invasion of BT549 cells (*p < 0.05, ***p < 0.001). [file 13046_2022_2260_MOESM7_ESM.tif]
