# Supplementary material for: Elevated expression of the rhythm gene NFIL3 promotes the progression of TNBC by activating NF-κB signaling through suppression of NFKBIA transcription
Source: J Exp Clin Cancer Res. 2022 Feb 18;41:67. doi: 10.1186/s13046-022-02260-1 (PMC8855542; doi:10.1186/s13046-022-02260-1)
Supplement: Supplementary file 8 — Additional file 8: Supplementary Table 1. [file 13046_2022_2260_MOESM8_ESM.docx]

**Supplementary Table 1**

| Primer name | Primer sequence (5’to 3’) |
| --- | --- |
| NFIL3-F | TCCTCAGTAGAACACACGCAG |
| NFIL3-R | TCATCTCTTGGCTCCCTTGT |
| NFKBIA-F | ACCTGGTGTCACTCCTGTTGA |
| NFKBIA-R | CTGCTGCTGTATCCGGGTG |
| Tubulin-F | ACCTTAACCGCCTTATTAGCCA |
| Tubulin-R | ACATTCAGGGCTCCATCAAATC |
| GAPDH-F | GACCTGACCTGCCGTCTA |
| GAPDH-R | AGGAGTGGGTGTCGCTGT |
| pLKO-NFIL3 #1282-F | CCAGAGAACTTGTATTTGAAG |
| pLKO-NFIL3 #1282-R | CTTCAAATACAAGTTCTCTGG |
| pLKO-NFIL3 #970-F | CCACACAAGCTCCGGATCAAA |
| pLKO-NFIL3 #970-R | TTTGATCCGGAGCTTGTGTGG |
| pLKO-NFKBIA #615-F | GGGTGCTGATGTCAATGCTCA |
| pLKO-NFKBIA #615-R | TGAGCATTGACATCAGCACCC |
| pLKO-NFKBIA #866-F | ATGACACAGAGTCAGAGTTCA |
| pLKO-NFKBIA #866-R | TGAACTCTGACTCTGTGTCAT |
| pLKO-NFKBIA #693-F | CCTGGTGTCACTCCTGTTGAA |
| pLKO-NFKBIA #693-R | TTCAACAGGAGTGACACCAGG |
| CRY2-F | CCACCGCAGCAGCAGAGTA |
| CRY2-R | CCAGCCCAGGCAACATTCA |
| PER1-F | TGGACAAAGTTGCTGACA |
| PER1-R | TCCCCGCAATAAATAAGT |
| NR1D1-F | CCTGGGAGTCTACAAGTGG |
| NR1D1-R | GATTGATGCGGACGATGGA |
| TIMELESS-F | CAGAACCTCACCAACTACTAT |
| TIMELESS-R | ACAGAATCGCTCCAACATT |
| EGR3-F | CACCTCACCACTCACATC |
| EGR3-R | CTTCTCCGCCTTCTTCTC |
| ChIP-NFIL3-F | TTGTCTTGTCGTGTCTGAA |
| ChIP-NFIL3-F | GCAGCGTTGGATATTAGATC |
| Luciferase-NFIL3-F | ATGAACGCGTTTGTCTTGTCGTGTCTGAA |
| Luciferase-NFIL3-R | AGTTCTCGAGGCAGCGTTGGATATTAGATC |
| ChIP-NFKBIA-F | GGGAGACCTGGCCTTCCTCAAC |
| ChIP-NFKBIA-R | GTGGGTCATAAAGACCTCACCAAATCA |
| Luciferase-NFKBIA-F | ATTATACGCGTGGGGTCAGGCTCGGGGAATT |
| Luciferase-NFKBIA-R | ACTAACTCGAGCCCTAACCACAGTGCGTCCTTC |
